# HIV-1 Unique Recombinant Forms Identified in Slovenia and Their Characterization by Near Full-Length Genome Sequencing

**DOI:** 10.3390/v12010063

**Published:** 2020-01-03

**Authors:** Maja M. Lunar, Jana Mlakar, Tomaž Mark Zorec, Mario Poljak

**Affiliations:** Institute of Microbiology and Immunology, Faculty of Medicine, University of Ljubljana, 1000 Ljubljana, Slovenia; maja.lunar@mf.uni-lj.si (M.M.L.); jana.mlakar@mf.uni-lj.si (J.M.); tomaz-mark.zorec@mf.uni-lj.si (T.M.Z.)

**Keywords:** HIV-1, molecular epidemiology, subtype, unique recombinant form, near full-length genome, next-generation sequencing, surveillance

## Abstract

Surveillance of HIV circulating recombinant forms (CRFs) is important because HIV diversity can affect various aspects of HIV infection from prevention to diagnosis and patient management. A comprehensive collection of *pol* sequences obtained from individuals diagnosed with HIV-1 from 2000 to 2016 in Slovenia was subtyped to identify possible unique recombinant forms (URFs). Selected samples were subjected to near full-length genome (NFLG) sequencing and detailed recombination analyses. Discordant subtyping results were observed for 68/387 (17.6%) sequences and 20 sequences were identified as the most probable URFs and selected for NFLG characterization. Further, 11 NFLGs and two sequences of >7000 base pairs were obtained. Seven sequences were identified as “pure” subtypes or already characterized CRFs: subtype B (*n* = 5), sub-subtype A6 (*n* = 1), and CRF01_AE (*n* = 1). The remaining six sequences were determined to be URFs; four displayed a single recombination event and two exhibited a complex recombination pattern involving several subtypes or CRFs. Finally, three HIV strains were recognized as having epidemic potential and could be further characterized as new CRFs. Our study shows that the identification of new CRFs is possible, even in countries where HIV diversity is considered limited, emphasizing the importance of the surveillance of HIV recombinant forms.

## 1. Introduction

Even though a successful antiretroviral treatment (ART) has been available for over two decades, human immunodeficiency virus (HIV) remains one of the major global disease burdens, causing 770,000 deaths in 2018 [1]. New HIV infections have decreased considerably since the peak in 1997, from 2.9 million to 1.7 million in 2018; however, we are still far from ending the epidemic and all measures need to be addressed to do so [1]. Two major hurdles are the viruses ability to integrate into the human genome and its high genetic diversity, hampering the extensive efforts to design a drug to eradicate HIV from its reservoirs and to design an effective prophylactic vaccine. Even if a vaccine becomes available and adopted, continuous surveillance of HIV subtypes and recombinant forms will remain crucial [2].

The major pandemic HIV-1 group M is represented by 10 subtypes (A–D, F–H, J, K, and the recently established subtype L) and 98 characterized circulating recombinant forms (CRFs), found in at least three epidemiologically unlinked individuals [3,4,5]. Some of the CRFs are so-called second-generation recombinants (SGRs), composed of two or more previously characterized CRFs [6]. A recent systematic review of all published and unpublished HIV-1 subtyping data collected between 1990 and 2015 showed that subtype C is currently responsible for almost half of HIV-1 infections globally, followed by subtype B and subtype A. The global prevalence of all recombinant forms was estimated at 22.8% between 2010 and 2015, with CRFs accounting for 16.7% and unique recombinant forms (URFs) for 6.1% of all global HIV-1 infections. A steady increase in the prevalence of recombinants over time has been noted [7]. HIV diversity can affect various aspects of HIV infection, from transmission and pathogenesis to diagnostic tests and viral load measurements, ART effectiveness and drug resistance, immune response, and vaccine development [6]. Thus, surveillance of circulating recombinant forms is considered essential.

The Slovenian HIV-1 epidemic is mainly driven by subtype B; however, 22.2%, 10.7%, and 17.3% of non-B subtypes were identified in Slovenia in the 2000–2004, 2005–2010, and 2011–2016 study periods, respectively [8,9,10]. In addition, 5.2% of CRFs (02_AG) and 7.8% of URFs in the 2000–2004 study period, 0.7% of CRFs and 2.0% of URFs in 2005–2010 period, and 2.4% of CRFs and 3.6% of URFs in the 2011–2016 study period were observed among the subtyped sequences [8,9,10].

In this study, we have characterized potential HIV-1 URFs identified in Slovenia by obtaining near full-length genome (NFLG) sequences and performing detailed recombination analyses.

## 2. Materials and Methods

A total of 634 persons were diagnosed with HIV-1 in Slovenia from 2000 to 2016. Among them, 387 (61.0%) partial *pol* sequences were obtained previously to study the prevalence of pre-treatment HIV-1 drug resistance in Slovenia as part of the European Commission—sponsored SPREAD program [8,9,10], approved by the national Medical Ethics Committee at the Slovenian Ministry of Health (approval reference number 126/12/03). This study was conducted in accordance with the Code of Ethics of the World Medical Association (Declaration of Helsinki) and all the patients gave consent to participate in the study.

Sequences were analyzed for evidence of recombination with the following subtyping tools: Rega HIV-1 & 2 automated subtyping tool, version 2.0 (Rega 2) [11], Rega HIV-1 subtyping tool, version 3.0 (Rega 3) [12], COMET HIV-1, version 1.0 (Comet) [13], jpHMM [14], SCUEAL [15], Geno2pheno [16], and Stanford HIVdb Program [17].

Next, phylogenetic analysis was performed for further inspection of Slovenian sequences that yielded at least one discordant subtyping result with the seven subtyping tools. These sequences were aligned together with the Compendium alignment which was obtained from the Los Alamos database (all subtypes and CRFs of group M available at that time and three sequences of group O to be used as the outgroup) and a set of the most similar sequences to serve as controls [18]. Control sequences were obtained from a Los Alamos database blast search, in which 10 sequences per each Slovenian sequence were downloaded [18,19]. Duplicated sequences were removed from the dataset and aligned by MUSCLE available in Mega version X [20]. Maximum likelihood phylogeny was inferred by employing PhyML with automatic model selection according to the Akaike information criterion [21]. Nearest neighbor interchange was employed as a tree improvement method and aLRT (approximate likelihood ratio test) was used to assess the significance of transmission clusters. The phylogenetic tree was visualized in Figtree, version 1.4.3 [22].

Samples were selected for further NFLG analyses according to the results of the subtyping tools and the phylogenetic analysis; specifically, if the sequences were positioned: (i) at the outskirts of subtype clusters or (ii) alongside previously determined URFs on the phylogenetic tree.

NFLG sequences were obtained according to the previously published protocol [23]. Briefly, four overlapping PCR amplicons were obtained and joined in equimolar amounts by using a Qubit dsDNA HS Assay kit (Thermo Fisher Scientific, Waltham, MA, USA). A library was prepared using Nextera DNA Flex and sequenced on an Illumina MiSeq platform (Illumina, San Diego, CA, USA). The FastQ files that were obtained were assembled with a SHIVER pipeline [24]. Briefly, contigs were assembled de novo from paired-end reads using IVA [25] and compared to the reference set of HIV sequences, Compendium HIV-1 genome alignment (2017), which were retrieved from the Los Alamos database using BLASTN [18,19]. Contigs representing HIV sequences were corrected and aligned to the references for visual inspection. Then, a mapping reference was created from the contigs and the gaps between the contigs were filled with the closest reference sequence. Reads were trimmed of primer and adapter sequences and of low-quality reads using Trimmomatic [26] and Fastaq [27] and then mapped to the compiled reference using Smalt [28]. Finally, a consensus was called using SAMtools [29].

The NFLG sequences that were obtained were first analyzed with the following subtyping tools: Rega 3, Comet, and jpHMM. SCUEAL can only be used to determine the subtype for *pol* sequences and was therefore not employed. Similarly, the primary aim of Geno2pheno and the Stanford HIVdb Program is genotypic HIV drug resistance interpretation and they are therefore designed to analyze genome regions targeted by antiretroviral drugs and were also not employed.

Further recombination analyses were carried out using SimPlot 3.5.1 [30]. The NFLG sequences that were obtained were first examined by performing exploratory similarity plot analyses available in SimPlot, with all HIV subtype reference sequences available from the SimPlot 3.5.1 Los Alamos HIV database [18]. Next, the bootscan method was employed with query sequences corresponding to subtypes A–D, F–H, J, K, and CRF01_AE or with a corresponding subset of subtype references. The bootscan procedure was run with 500 bootstrap replicates and a 400 base-pair (bp) sliding window with a 10 bp step size, without gap stripping. If the sequence was suspected to be a secondary recombinant (with recombination among CRFs), CRFs that were identified in a similarity plot were also included in the bootscanning. Finally, recombination breakpoints were identified and slices of the alignments were saved for further phylogenetic analyses, performed as described above. Final unique recombinant forms were visualized with the Recombinant HIV-1 Drawing Tool [31].

An additional BLAST search of NFLG sequences determined to be URFs was employed to search for sequences with the highest identity based on the *pol* region. The BLAST hits that were obtained were further analyzed with the selected subtyping tools, which displayed a recombination signal when query URF sequences were first analyzed [19]. If two or more sequences were identified with a similar recombination pattern, the URF was identified as a candidate for a new CRF.

## 3. Results

### 3.1. Subtyping of Pol Region

The subtyping tools yielded at least one discordant result in 68/387 (17.6%) partial *pol* sequences obtained from individuals with an HIV-1 diagnosis from 2000 to 2016 in Slovenia (Table 1). The most discordant results were obtained with Rega 2 (*n* = 32), followed by Rega 3 (*n* = 27), Geno2pheno (*n* = 23), SCUEAL (*n* = 21), Stanford (*n* = 21), jpHMM (*n* = 12), and Comet (*n* = 11). On the other hand, the subtyping tool that gave an indication of a possible URF in the most cases was, again, Rega 2 (*n* = 28), followed by Rega 3 (*n* = 22), SCUEAL (*n* = 21), Stanford (*n* = 20), jpHMM (*n* = 13), and Comet (*n* = 11). Geno2pheno did not report any cases of possible recombination (Table 1).

**Figure 1:**
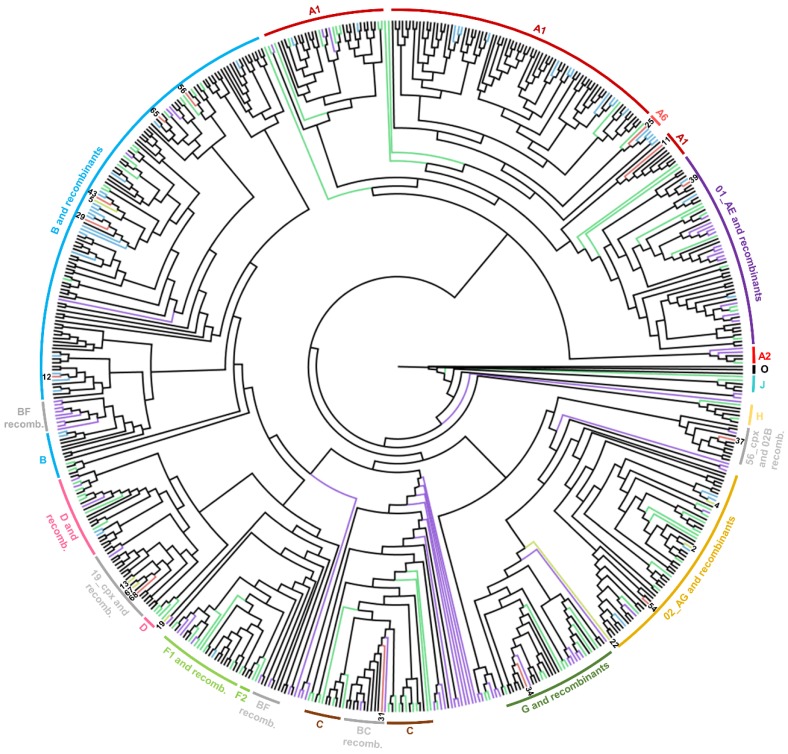
Maximum likelihood phylogenetic tree of the *pol* region of the divergent Slovenian sequences. Sample numbers selected for further near full-length genome characterization are depicted at the tips of the branches, which are colored red if the near full-length genome sequence was obtained, yellow if the sequencing was not successful, and blue if it was not initially selected for sequencing. Subtype reference sequences of subtypes A–D, F–H, J, K, CRF01_AE, and CRF02_AG are depicted in green, other CRF references are shown in purple, and other control sequences are in black.

All 68 potential recombinant sequences were included in the phylogenetic analysis together with 365 control sequences, all available reference sequences of HIV-1 group M, and three sequences of HIV-1 group O as the outgroup. The alignment was carried out on 614 sequences altogether (positioning according to the HXB2 reference sequence: 2268–3269 bp) with a length of 1002 bp, and phylogeny was inferred (Figure 1). According to the results of the subtyping tools and the *pol* phylogenetic analysis, 20 sequences were identified as the most probable unique recombinant forms and were selected for further NFLG characterization (Figure 1, Table 1).

### 3.2. Near Full-Length HIV Genome Sequencing and Characterization

We successfully obtained all four overlapping amplicons for 13/20 (65%) of the samples, which were afterwards sequenced using the Illumina MiSeq platform. Due to low read coverage of the fourth PCR amplicon, the expected length of the NFLG sequence was not obtained in two cases and ended at nucleotide positions HXB2: 7842 bp and HXB2: 7843 bp, respectively.

**Figure 2:**
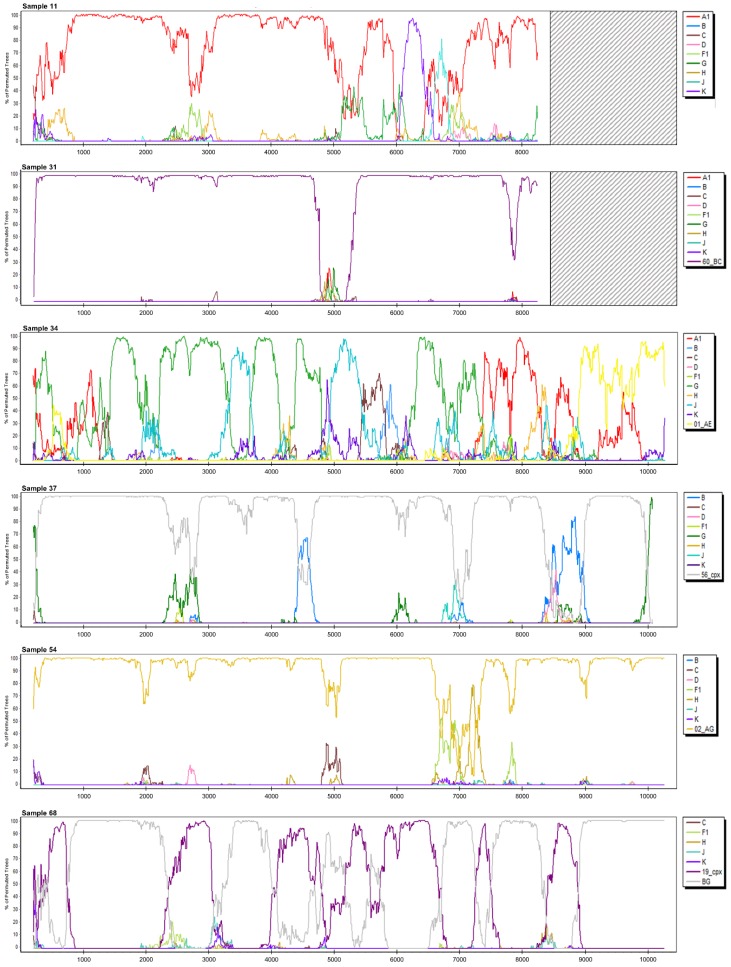
SimPlot bootscan analysis of the Slovenian near full-length HIV-1 genome sequences characterized as recombinants, where the *x*-axis represents the nucleotide position in the alignment.

**Figure 3:**
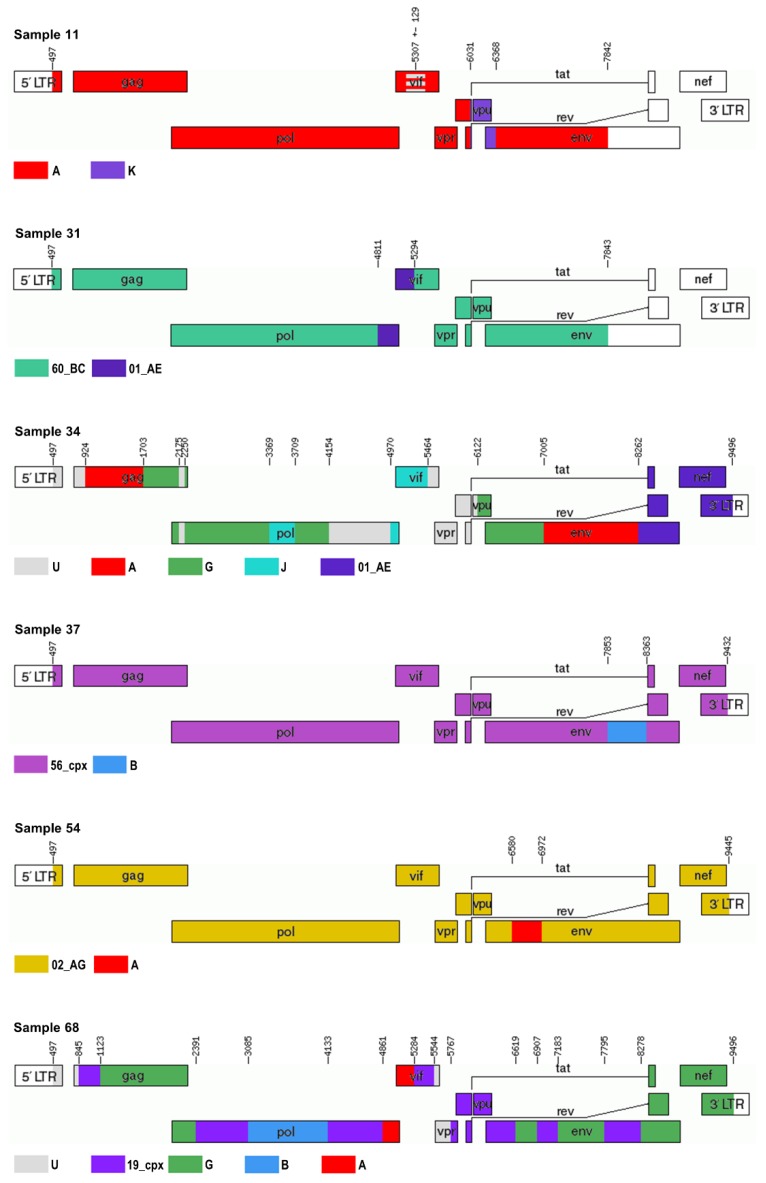
Final unique recombinant forms visualized with the Recombinant HIV-1 Drawing Tool, where recombination breakpoints are depicted according to nucleotide position in the reference HXB2.

The NFLG sequences obtained were first analyzed with Rega 3, Comet, and jpHMM (Table 2). Second, an exploratory similarity plot analysis and bootscanning, available in SimPlot, were performed (Table 2, Figure 2 and Figure S1). Recombination breakpoints were identified and slices of the alignments were saved for further phylogenetic analyses. Subtypes/CRFs were assigned and possible unique recombinants were characterized in seven and six cases, respectively (Table 2, Figure 3).

To observe the sensitivity and specificity of subtyping tools based on the *pol* region, we inspected the subtyping results among the six sequences determined to be URFs. SCUEAL performed best, with five results indicative of recombination, followed by Rega 2 (*n* = 4) and jpHMM (*n* = 4). Rega 3, Comet, and Stanford all detected recombination in half of the cases, whereas Geno2pheno did not detect them at all. On the other hand, a false-positive recombination signal in the sequences, characterized as non-recombinant by NFLG analysis, was detected with Rega 3 (5/7), Rega 2 (4/7), and SCUEAL (1/7).

No clinically relevant drug resistance mutations were identified in the obtained NFLG sequences by the Stanford HIVdb Program.

#### 3.2.1. Sample 11

The sequence of 7320 bp obtained was determined to be subtype A1/K recombinant with jpHMM, whereas Comet and Rega 3 assigned subtype A1 to it. SimPlot analysis characterized it as A1/K recombinant or CRF45_cpx (Table 2, Figure 2). Phylogenetic analysis was performed on nine separate slices of alignment and displayed clustering with subtype A in 7/9 occurrences with aLRT values of >0.85 (Figure S2). Phylogenetically, the closest sequence was CRF45_cpx in 3/9 segments, followed by subtype A1, CRF09_cpx, and CRF63_02A in one segment each. The remaining three alignments of the query sequence did not cluster within transmission pairs. Alignment slice HXB2: 5178–5436 bp clustered within the subtype A cluster, but could not be distinguished from CRF01_AE, and thus, this region was designated as an area of less certainty (A/unclassified (U)). Slice HXB2: 6031–6367 bp clustered with subtype K sequences (aLRT = 0.93). Both partitions clustered in a transmission pair with CRF45_cpx (Figure S2). Finally, Sample 11 was characterized as a URF of subtypes A and K with an area of uncertainty at position HXB2: 5307 ± 129 bp (Figure 3).

The sample was obtained in 2008 from an individual who reported that he most likely acquired HIV from a male of African origin residing in Slovenia. A BLAST search of the *pol* region revealed another similar Slovenian sequence without a known epidemiological link and several sequences from Nigeria and Cameroon. It is apparent that this variant is composed of subtype K and of a divergent subtype A1, as shown in Figure 1, which is possibly related to the ancestor of CRF01_AE. A highly significant phylogenetic cluster (aLRT = 0.99) encompassing the Sample 11 sequence and several sequences from Africa was observed on the *pol* phylogenetic tree. Thus, this variant could be a candidate CRF when two additional NFLGs obtained from unlinked individuals are sequenced.

#### 3.2.2. Sample 12

We obtained a sequence of 9014 bp and the three subtyping tools determined it to be of subtype B (Table 2). This was confirmed by the detailed SimPlot analysis; however, two regions exhibiting less significant bootstrap support with subtype B were identified (HXB2: 3797–4218 bp and 8882–9183 bp; Figure S1). Further phylogenetic analyses of the two segments confirmed clustering within subtype B sequences with aLRT of 0.73 and 0.96, respectively.

#### 3.2.3. Sample 25

We successfully sequenced 9053 bp of the HIV genome. The three subtyping tools determined this sequence to be of sub-subtype A1; however, SimPlot analyses determined it to be of sub-subtype A6 (Table 2, Figure S1). The sample was obtained from a person originating from Ukraine. Three additional individuals were identified with this subtype in Slovenia, according to the *pol* phylogenetic tree.

#### 3.2.4. Sample 29

We sequenced 8964 bp of the genome and assigned it subtype B by the subtyping tools as well as SimPlot analyses (Table 2, Figure S1).

#### 3.2.5. Sample 31

Only 7308 bp were obtained due to poor read coverage of the fourth PCR amplicon. jpHMM and Comet determined the sequence to be a complex recombinant, whereas Rega 3 assigned subtype C to it. SimPlot analysis suggested it to be CRF60_BC with an area of poor bootstrap support of the segment HXB2: 4811–5293 bp (Figure 2). This region was identified as CRF01_AE by phylogenetic analysis with aLRT of 0.76 (Figure S2). The surrounding two regions were confirmed as CRF60_BC with aLRT of 1.00 and therefore, this sequence was proposed to be a URF consisting of CRF60_BC and CRF01_AE (Table 2, Figure 3).

A BLAST search identified sequences of the recently characterized Italian CRF60_BC recombinant as sequences with the highest identity scores [32]. The phylogenetic tree of the *pol* region displayed a Sample 11 sequence on the root of the cluster of these CRF60_BC sequences from Italy, with aLRT = 1.00. Thus far, we do not have data for additional samples with this CRF60_BC and CRF01_AE recombinant and therefore, we do not have any indications that this variant is still circulating and we propose that it is a unique recombinant.

#### 3.2.6. Sample 34

We sequenced 9030 bp of the genome and all three subtyping tools identified it as a complex recombinant (Table 2). This was also evidenced in the further SimPlot analysis, which indicated a recombinant of subtypes A, G, J, C, and CRF01_AE (Figure 2). Namely, 17 separate slices of alignment were saved according to bootscanning and corresponding phylogenetic analyses were performed (Figure S2). A subtype could not be assigned to seven segments; however, the remaining segments were assigned subtypes A1, G, J, and CRF01_AE. CRF13_cpx was found to be most closely related to this sequence on 5/17 phylogenetic trees, followed by CRF11_cpx (3/17; Figure S2). Finally, a complex URF of subtypes A, G, J, CRF01_AE, and U was described, as shown in Table 2 and with HXB2 breakpoints in Figure 3.

The individual originates from Gambia and a BLAST search revealed the sequence to be most similar to another Slovenian sequence obtained from a person with an epidemiological link, as we know from the patient’s records. Other similar sequences were from Liberia and Nigeria; however, those sequences did not show the same recombination signal when the *pol* region was analyzed and therefore, we do not have data that more sequences are circulating and we determined that this variant is a URF and not a CRF candidate.

#### 3.2.7. Sample 37

The NFLG sequence of 8979 bp displayed a complex recombination pattern with all three subtyping tools (Table 2). SimPlot proposed a recombinant of CRF56_cpx and subtype B. Five different segments of the alignment were generated and phylogenetic analysis confirmed CRF56_cpx as the most closely related sequence in 4/5 segments, whereas subtype B was determined to be the origin of the remaining segment of the genome (HXB2: 7853–8362 bp; Figure S2). CRF56_cpx clustered with 02_AG sequences on the phylogenetic tree of this segment (Figure S2).

A BLAST search identified several sequences from Turkey, displaying high identity. This was also observed on the *pol* phylogenetic tree, where the query sequence was in a transmission cluster with Turkish sequences (aLRT = 0.99). Comet subtyped the most similar sequence as an unassigned sequence composed of CRF02_AG, CRF56_cpx, and subtype B, and therefore it is possible that this recombinant is in circulation and could thus be a CRF candidate.

In addition, we obtained this sample in 2012 from a person who reported that he most probably acquired HIV in Turkey.

#### 3.2.8. Sample 39

Comet detected possible recombination between CRF01_AE, subtype B, and subtype D in the sequence of 9048 bp; however, Rega 3 and jpHMM identified it as CRF01_AE. SimPlot analysis showed high bootstrap support for CRF01_AE throughout the sequence and thus, we concluded that this sequence is CRF01_AE (Figure S1).

#### 3.2.9. Sample 43

Only Comet identified this sequence of 8910 bp as a potential recombinant of subtypes B, C, and G, whereas jpHMM and Rega 3 determined it to be subtype B (Table 2). SimPlot, on the other hand, displayed three regions of low bootstrap support with subtype B and indicated a recombinant of subtype D (Figure S1). Two of the segments were clearly within the subtype B cluster on the phylogenetic tree. For the last segment (HXB2: 8929–9412 bp), the subtype could not be determined, possibly due to the high similarity between subtype B and subtype D because subtype D sequences were nested within the subtype B cluster. We could not confirm any recombination event in this sequence and determined it to be of subtype B (Table 2).

#### 3.2.10. Sample 54

Rega 3 and Comet subtyped the sequence of 8970 bp as CRF02_AG and jpHMM as subtype A1/G recombinant (Table 2). Bootscan identified it as CRF02_AG with two sections of lower bootstrap support, which were further analyzed phylogenetically (Figure 2 and Figure S2). Segment HXB2: 6580–6971 bp clustered with sub-subtype A1 sequences (aLRT = 0.87), distinctly separated from the CRF02_AG cluster (Figure S2). This sequence was therefore characterized as CRF02_AG and subtype A recombinant (Figure 3).

The sequence with the highest identity retrieved from a BLAST search was a sequence from Cameroon (MH705136) and was subtyped as CRF02_AG. Therefore, we do not have any evidence to suggest that this variant is still in circulation and so we propose that it is a URF.

#### 3.2.11. Sample 56

A sequence of 9010 bp was obtained and identified as subtype B by the three subtyping tools as well as SimPlot analysis (Table 2, Figure S1).

#### 3.2.12. Sample 65

The sequence of 9039 bp obtained was determined to be subtype B by the subtyping tools and SimPlot (Table 2, Figure S1).

#### 3.2.13. Sample 68

The NFLG sequence of 9048 bp was recognized as a complex recombinant by the three subtyping tools. Bootscan and SimPlot displayed the highest similarity with CRF19_cpx and subtype B/G recombinants (the group of CRF20_BG, CRF23_BG, and CRF24_BG). Separate phylogenetic analyses were performed on 15 sections of the alignment (Figure S2). Query sequence was found in a transmission pair with CRF19_cpx in 5/15 segments, in a cluster with CRF19_cpx and CRF37_cpx in two segments, and as a parental strain to CRF19_cpx in another segment. In another 5/15 segments, the sequence was observed in a phylogenetic cluster with subtype B/G recombinants, mostly in a close relationship with CRF20_BG (3/5). In one segment, phylogenetic analysis determined a close relationship with sub-subtype A1, and in two other segments, a decision could not be made and it was therefore determined to be U (Figure S2). Because we could not assign a clear BG recombinant for the non-CRF19_cpx regions of the genome, we propose that the recombinant is probably between CRF19_cpx and a still-undescribed subtype B/G/A recombinant and we have characterized the sequence as a recombinant of CRF19_cpx, subtype G, subtype B, subtype A, and U (Table 2, Figure 3).

The sequence was obtained in 2016 from a person of Slovenian origin who reported that he probably acquired HIV in Slovenia. A BLAST search showed the sequence to be most similar to two Slovenian sequences. The first was collected in 2008 from an individual who reported that he acquired the HIV infection in Italy. The second was collected in 2016. For now, we do not have data on whether these three sequences were obtained from epidemiologically linked individuals, apart from them being men who have sex with men (MSM). Another sequence exhibiting high identity was a sequence collected in 2008 in Spain (GU830956) and submitted under an unpublished study called “Identification of a new HIV-1 circulating recombinant form in Spain.” These four sequences formed a phylogenetic cluster (aLRT = 0.996) nested within the CRF19_cpx cluster on the phylogenetic tree of the *pol* region (Figure 1). Thus, this variant seems to have spread between countries, possibly continents, and it is therefore a good candidate for a new CRF.

## 4. Discussion

A comprehensive collection of sequences obtained from individuals diagnosed with HIV-1 from 2000 to 2016 in Slovenia was subtyped to study possible URFs. Discordant subtyping results were observed for 18% (68/387) of sequences and 20 samples were selected for NFLG sequencing. Eleven complete NFLGs and an additional two sequences of over 7000 bp of HIV-1 possible unique recombinants were obtained and characterized in this study. Seven sequences were identified as “pure” subtypes or already characterized CRFs: subtype B (*n* = 5), sub-subtype A6 (*n* = 1), and CRF01_AE (*n* = 1). The remaining six sequences were determined to be unique recombinants. Among these, four displayed a single recombination event between either “pure” subtypes or CRFs and three were so-called SGRs (CRF60_BC|CRF01_AE|CRF60_BC, CRF56_cpx|B|CRF56_cpx, and CRF02_AG|A|CRF02_AG). On the other hand, two sequences exhibited a complex recombination pattern involving several subtypes or CRFs as well as regions of unknown origin. A search for additional sequences with a similar recombination profile revealed that additional samples of unlinked individuals could be sequenced for three of the URFs identified and could therefore lead to the characterization of new CRFs.

The HIV epidemic in Slovenia is mainly driven by subtype B; therefore, it is not surprising that 25 of the 68 divergently subtyped *pol* sequences were subtyped as potential recombinants of subtype B by the subtyping tools. Five of the samples were subsequently sequenced and no apparent recombination was determined in the NFLG sequences—all sequences were determined to be subtype B.

An additional two samples were also subsequently not characterized as unique recombinants (CRF01_AE and sub-subtype A6). Interestingly, Sample 25 was chosen because it clustered in a divergent A1 clade on the *pol* phylogenetic tree, together with the sub-subtype A6 reference sequence. It had a discordant subtyping score because Stanford and Geno2pheno both subtyped it as CRF01_AE. However, all of the subtyping tools designed specifically for subtyping characterized it as sub-subtype A1 because sub-subtype A6 is not included in their reference alignments. Thus, none of the subtyping tools were able to correctly assign sub-subtype A6. This sub-subtype is still the predominant sub-subtype in Russia, hence its former name subtype A_FSU_ [33]. Sub-subtype A6 was shown to have the most recent common ancestor (tMRCA) around 1984 in the city of Odessa, Ukraine, from where it most likely spread to other cities of Ukraine and other FSU countries [34]. We obtained Sample 25 from a person who most probably acquired HIV in Ukraine. Forward transmission in Slovenia was observed, with three additional sequences identified.

We employed a subsequent BLAST search of those sequences recognized as potential URFs to look for other related sequences. It was carried out on the *pol* region only because of the most comprehensive database and it is therefore possible that some similar sequences were overlooked. We identified Sample 68 as having the most potential for a new CRF. This variant was detected in three individuals from Slovenia and it could have originated in Italy, based on an individual report of one patient. Another highly similar sequence was obtained in Spain. We determined it to be a recombinant of CRF19_cpx and most probably of a still-undescribed B/G/A recombinant, similar to that of CRF20_BG. We can speculate that the recombination occurred in Cuba and spread from there, based on the origin of CRF19_cpx, CRF20_BG, CRF23_BG, and CRF24_BG. A high prevalence of CRF19_cpx and CRF20, 23, 24_BG was observed among HIV-positive Cuban adults in a recent study: 24% and 23%, respectively [35]. This makes recombination events more plausible. On the other hand, CRF19_cpx spread was observed in southern Spain, where several transmission networks of young native Spanish MSM were determined, with tMRCAs in the period from 2007 to 2010 [36,37,38]. This, coupled with the recent characterization of new recombinant forms of subtypes B and G described in Spain and Portugal, could suggest that the URF identified in this study might also have originated in Europe [39,40].

Worryingly, CRF19_cpx has been associated with rapid disease progression among Cuban patients. Patients had a higher HIV viral load at diagnosis, they presented with AIDS within three years, and the variant was observed to be more frequently CXCR4-tropic and had better fitness of the protease [41]. We do not have data regarding disease progression for all three Slovenian individuals infected with this strain because two were diagnosed in 2016 and started treatment shortly after diagnosis, following the European HIV treatment guidelines [42]. On the other hand, the person diagnosed in 2008 was certain to have acquired HIV infection in Italy in 2007. He had a CD4 cell count of 445 cells/mm^3^ at diagnosis, three months later his CD4 cell count dropped to 351 cells/mm^3^, and afterward, antiretroviral treatment was initiated. This could indicate rapid disease progression, as mentioned above. Indeed, the protease region of Sample 68 partially consisted of CRF19_cpx.

Another potential candidate for CRF characterization is Sample 11. Five of the nine genome segments were observed in a close phylogenetic relationship with CRF09_cpx and/or CRF45_cpx. A study examining the evolutionary history of these complex recombinants found that CRF09_cpx and CRF45_cpx were positioned at the root of subtype A/A1 on the phylogenetic tree of the *env* region. This indicates that these two CRFs originate from variants that diverged from the ancestral lineage at a similar time as the ancestor of subtype A. Thus, the tMRCA of the *env* region was estimated to be around 1965 and 1960 for CRF09_cpx and CRF45_cpx, respectively. The tMRCA of the *pol* region was estimated to be a decade later [43]. The sequence of Sample 11 shows a less complex structure and yet the subtype A part of the sequence, which corresponds to the *env* part of that study, shows a high similarity to that of CRF09_cpx and CRF45_cpx. Similarly, we observed that these sequences clustered basally at the root of CRF01_AE on the *pol* phylogenetic tree. Interestingly, a NFLG sequence collected in 2010 in Nigeria (KX389608) was found among the clustered sequences. It was subtyped as subtype A by the authors; however, our jpHMM analysis displayed a nearly identical recombination profile to that of the Sample 11 sequence, with additional complexity at the 3′-end, which we failed to sequence. We obtained the Sample 11 sequence from a person who probably acquired the infection from an HIV-positive person of African origin and we identified another similar sequence from Slovenia and several African sequences. We can speculate that the variant is an old lineage that is still in circulation and it could be one of the progenitors of other CRFs.

Sample 37 was characterized as a CRF56_cpx|subtype B|CRF56_cpx recombinant. We identified several sequences with high similarity from Turkey, where the person whose sample was obtained also reported having been infected with this particular strain. Interestingly, CRF56_cpx was first described among young MSM from France. The tMRCA of around 2007 was estimated for this CRF, suggesting a recent diffusion. The authors described related strains found in Cyprus and Greece, and they proposed that they probably share a common ancestor [44]. This falls in line with our findings, implying that the origin of these strains could have been in the eastern Mediterranean region. In addition, CRF56_cpx has been observed in Pakistan and the Philippines, thus reaching global diffusion [45].

Sample 31 was recognized as a recombinant of CRF60_BC and CRF01_AE. The sequence was obtained from a person who reported having acquired HIV in Slovenia. We do not have further evidence that this variant has epidemic potential. However, a few SGRs composed of CRF60_BC have been described in Italy, where the CRF60_BC epidemic grew exponentially between 2006 and 2011 [32]. Because the CRF01_AE section of the Sample 31 sequence is outside the routinely genotyped *pol* region for resistance detection, further detection of this URF will be more challenging.

A complex recombination pattern was observed in the Sample 34 sequence, probably originating from Gambia. We could not assign a subtype to several regions of the sequence that was obtained. This indicates that these parts of the sequence are too divergent from the known subtypes and therefore this could represent a recombinant of an ancestral lineage or a lineage that subsequently died out. In fact, the sequence was found to be related in parts to CRF13_cpx and CRF11_cpx. These CRFs were shown to probably originate from Central Africa, where they emerged in the late 1950s to late 1960s [43]. Tongo et al. (2015) proposed that parts of genomes of the unique recombinant sequences from Cameroon that clustered outside of all identified subtypes and CRF clades are in fact historical lineages that diverged early in the HIV epidemic but did not reach the magnitude of an epidemic like other subtypes did [46].

The main limitation of this study is the selection of samples for URF characterization, which was based on subtyping and phylogenetic analysis of the *pol* region only, therefore possibly missing URFs that do not display recombination breakpoints within this region. However, the *pol* region was employed because the sequences were available from previous studies analyzing transmitted drug resistance in Slovenia [8,9,10]. Most Western European countries perform routine baseline drug resistance testing based on the *pol* region before treatment initiation and therefore, these generated sequences could additionally be employed for surveillance of HIV subtypes and CRFs and detection of emerging new CRFs with possible implications for HIV prevention, diagnosis, and management of HIV-positive individuals [7].

Seven different subtyping tools were employed for the initial subtyping of the *pol* region. The principal role of five of the aforementioned tools is the determination of the HIV-1 subtype; however, Stanford and Geno2pheno are used foremost for genotypic drug resistance interpretation, yet do allow for additional information of HIV-1 subtype. The two were included to compare the results with other subtyping tools and indeed Geno2pheno did not report any evidence of recombination, probably because this tool was not designed to do so in the first place. The Stanford algorithm showed recombination in half of the URFs subsequently characterized by NFLG sequencing and was thus more in line with other more complex subtyping algorithms. Because all the subtyping tools available seem to have some limitations, a combination of tools should preferably be used when looking for recombination.

Unfortunately, we were not able to obtain all four amplicons for seven of the samples selected for NFLG sequencing using the protocol by Gall et al. (2012) [23]. This protocol faces some problems, especially when older samples with a lower viral load were processed. For example, Sample 19 was detected in a transmission pair with CRF90_BF and Sample 22 was observed basal to the CRF02_AG cluster on the phylogenetic tree of the *pol* region and therefore, both were indicated for NFLG sequencing. Sadly, we could not obtain all the PCR products and further analyze these two samples of interest. In addition, read coverage of the fourth amplicon was too low for two samples and we were therefore not able to obtain the complete sequences.

The highest HIV-1 genetic diversity was observed in the Democratic Republic of Congo, where the HIV-1 epidemic was shown to originate [47,48]. Due to error prone reverse transcriptase enzyme and high replication rates, HIV has the ability to generate genetically diverse viral variants within a single individual that can differ by up to 10% [49,50,51]. Variation within a subtype and between subtypes is even greater and can be as high as 30% and 42% at the amino acid level, respectively [6,51]. That is why the HIV pandemic is characterized by the remarkable global genetic diversity, reflected in the abundance of HIV subtypes and recombinant forms circulating in the world. Apart from HIV subtypes, several CRFs have obtained epidemic proportions and have become drivers of the HIV epidemic in various regions, such as CRF01_AE in Southeast Asia and CRF02_AG in West Africa. Furthermore, CRFs and URFs are continuously increasing in prevalence [7]. However, what specific characteristics of CRFs contribute to their dispersal is still to be elucidated. Propagation of a newly generated URF into a CRF with epidemic significance is probably a result of a mosaic of factors. Genetic characteristics of the virus itself and immunological traits of the population at risk of infection both contribute to transmissibility and disease progression [7,52,53]. These biological properties coupled with socioeconomic factors, such as transportation links, migration, population growth, access to treatment, and to some extent, stochastic effect, all add to the effective spread of a particular viral variant [7,48,54,55].

Since HIV diversity plays a central role in the HIV pandemic, today it is imperative that global molecular epidemiology surveillance is continued and improved using rigorous sampling strategies in different parts of the world. With the implementation of next-generation sequencing in smaller-scale laboratories, this technique allows identification of new CRFs, even in countries where HIV diversity is substantially limited, like Slovenia. For this reason, Rodgers et al. (2017) propose making changes to HIV-1 nomenclature and suggest that it should only include CRFs of epidemiological significance [47]. Indeed, as mentioned above, it is difficult to predict which of the CRFs identified will reach epidemic potential, thus their surveillance is of utmost importance. For example, CRFs such as the previously mentioned CRF19_cpx and CRF14_BG were associated with rapid disease progression to AIDS and death, suggesting high pathogenicity and virulence of these strains [41,56]. In addition, HIV variants can differ in their rate of transmission and can influence the sensitivity of diagnostic tests and viral load measurements and the effectiveness of ART and vaccines, highlighting the importance of continuous surveillance of circulating recombinant forms [6].

## 5. Conclusions

Six unique recombinant forms were identified among individuals diagnosed with HIV-1 between 2000 and 2016 in Slovenia, and three HIV strains were recognized as having epidemic potential and could be further characterized as new CRFs. Our study shows that the identification of new CRFs is possible, even in countries where HIV diversity is limited, emphasizing the importance of the surveillance of HIV-1 recombinant forms.

## Figures and Tables

**Table 1:** Subtyping results of sequences that had at least one divergent result with the seven subtyping tools. Samples subsequently selected for further near full-length genome sequencing according to phylogenetic analysis are shown in bold.

No.	Accession Number	Rega 2.0	Rega 3.0	Comet 1.0	jpHMM	SCUEAL	**Stanford**	**Geno2pheno 3.3**
1	AJ971094	NA	02_AG	02_AG	A1, G	02_AG-like	02_AG/02_AG	02_AG
**2**	**AJ971095**	**NA**	**02_AG**	**02_AG**	**A1, G**	**02_AG-like/complex**	**02_AG/02_AG**	**02_AG**
3	AJ971100	F (F1)	F (F1)	F1	F1	F1	D/F	F1
**4**	**AJ971096**	**NA**	**02_AG**	**02_AG**	**A1, G**	**02_AG-like**	**02_AG/02_AG**	**02_AG**
**5**	**AJ971133**	**NA**	**B-like**	**B**	**B**	**B, D recombinant/B**	**B/B**	**B**
6	GQ399318	B	Recombinant of B, D	B	B	B	B/B	B
7	AM113750	NA	02_AG	02_AG	A1, G	complex	02_AG/02_AG	02_AG
8	JX046417	A (A1)	A (A1)	A1	A1	A-ancestral, A1 rec/A, A1 recombinant/A1	A/CRF01_AE	01_AE
9	JX046416	A (A1)	A (01_AE)	01_AE	01_AE	AE	01_AE/01_AE	01_AE
10	JX046415	NA	02_AG	02_AG	A1, G	complex	02_AG/02_AG	02_AG
**11**	**JX046413**	**A (A1)**	**A (A1)**	**A1**	**A1**	**complex/A, A-ancestral recombinant**	**A/01_AE**	**01_AE**
**12**	**JX028352**	**B**	**NA (recombinant of B, D)**	**B**	**B**	**B**	**B/B**	**B**
**13**	**JX046414**	**19_cpx**	**19_cpx**	**unassigned; D, 11_cpx, G, 20_BG**	**D**	**complex**	**D/D**	**D**
14	JX028338	NA	B	B	B	B	B/B	B
15	JX046406	D	D	D	B, D	D	D/D	D
16	JX046405	D	D	D	B, D	D	D/D	D
17	JX028323	NA	B	B	B	B	B/B	B
18	JX046407	NA	B, potential recombinant	B	B	B	B/B	B
**19**	**JX046408**	**NA**	**Recombinant of F1, 05_DF**	**unassigned; B, F1**	**B, F1**	**B, F1 recombinant**	**B/F**	**F1**
20	JX046409	A (A1)	A (A1)	A1	A1	A1	A/01_AE	01_AE
21	JX046410	A (A1)	A (A1)	A1	A1	A1	A/01_AE	01_AE
**22**	**KP013668**	**G**	**G**	**unassigned; 18_cpx, 02_AG, G**	**G**	**G**	**G/G**	**02_AG**
23	JX046412	A (A1)	A (A1)	A1	A1	A1	A/01_AE	01_AE
24	JX046404	A (A1)	A (A1)	A1	A1	A1	A/01_AE	01_AE
**25**	**JX046411**	**A (A1)**	**A (A1)**	**A1**	**A1**	**A1**	**01_AE/01_AE**	**01_AE**
26	KP013669	A (A1)	A (A1)	A1	A1	A1	A/01_AE	01_AE
27	JX028308	B	NA, Recombinant of B, D	B	B	B	B/B	B
28	JX046403	A (A1)	A (A1)	A1	A1	A1	A/01_AE	01_AE
**29**	**KF753740**	**NA**	**Recombinant of B, D**	**B**	**B**	**B**	**B/B**	**B**
30	KF753737	NA	Recombinant of B, D	B	B	B	B/B	B
**31**	**KF753747**	**NA**	**C, potential recombinant**	**unassigned; F1, A1, 01_AE, B, C, 60_BC**	**A1, B, C**	**complex**	**C/D**	**C**
32	KF753751	A (A1)	A (A1)	A1	A1	A1	01_AE/01_AE	01_AE
33	KF753741	A (A1)	A (A1)	A1	A1	A1	A/01_AE	01_AE
**34**	**KF753731**	**G**	**G**	**unassigned; D, J, G**	**G, J**	**AE, G recombinant**	**A/G**	**G**
35	KF753711	B	B	unassigned; B-D	B	B	B/B	B
36	KP013665	B	B	B	B	B, D recombinant/B	B/B	B
**37**	**KF753704**	**NA**	**NA (G, B)**	**G (check for 02_AG)**	**A1, B, G**	**complex**	**02_AG/B**	**02_AG**
38	KF753709	NA	B	B	B	B	B/B	B
**39**	**KF753699**	**A (A1)**	**A (01_AE)**	**01_AE**	**01_AE**	**15_01B/A1, AE recombinant/AE, F-ancestral recombinant**	**01_AE/01_AE**	**01_AE**
40	KP013656	B	B	B (check for 29_BF)	B	B	B/B	B
41	KP013648	A (A1)	A (A1)	A1	A1	A1	01_AE/A	A1
42	KP013643	NA	B-like	B	B	B	B/B	B
**43**	**KP013657**	**NA**	**Recombinant of B, D**	**B**	**B**	**B**	**B/B**	**B**
44	KP013641	A (A1)	A (A1)	A1	A1	A1	A/01_AE	01_AE
45	KP013660	A (A1)	A (A1)	unassigned; D, 10_CD, 01_AE, B	01_AE	A1, AE recombinant	01_AE/01_AE	01_AE
46	KP013644	NA	B	B	B	B	B/B	B
47	KY656620	B	B-like	B	B	B	B	B
48	KY656621	NA	B-like	B	B	B	B	B
49	MN736711	B	B	B	B	B, C recombinant	B	B
50	KY656626	A (A1)	A (A1)	A1	A1	A1	A (A_FSU)	01_AE
51	KY656630	NA	02_AG	02_AG	A1, G	A4, G recombinant	02_AG	02_AG
52	KY656636	A (A1)	A (A1)	A1	A1	A1	A (A_FSU)	01_AE
53	KY656616	NA	B-like	B	B	B	B	B
**54**	**KY656639**	**NA**	**G (02_AG)**	**02_AG**	**F1, G**	**G**	**02_AG**	**02_AG**
55	KY656643	A (A1)	A (A1)	A1	A1	A1	A	01_AE
**56**	**KY656648**	**NA**	**Recombinant of B, D**	**B**	**B**	**B**	**B**	**B**
57	KY656649	B	B	B	B	B, F1 recombinant	B	B
58	KY656650	B	Recombinant of B, D	B	B	B	B	B
59	KY656640	A (01_AE)	A (01_AE)	01_AE (check for 15_01B)	01_AE	AE	01_AE	01_AE
60	KY656656	NA	B-like	B	B	B	B	B
61	KY656657	A (A1)	A (A1)	A1	A1	A1	A, 01_AE	01_AE
62	KY656658	A (A1)	A (A1)	A1	A1	A1	A, 01_AE	01_AE
63	KY656659	A (A1)	A (A1)	A1	A1	A1	A, 01_AE	01_AE
64	MF987697	NA	Recombinant of B, D	B	B	B	B	B
**65**	**KY656670**	**NA**	**Recombinant of B, D**	**B**	**B**	**B**	**B**	**B**
66	KY656671	A (A1)	A (A1)	A1	A1	A1	A/01_AE	01_AE
**67**	**MF987719**	**NA**	**NA**	**unassigned, B–G**	**D**	**complex**	**D**	**D**
**68**	**MF987720**	**NA**	**NA**	**unassigned, B–G**	**D**	**complex**	**D**	**D**

NA = not assigned; FSU = former Soviet Union.

**Table 2:** Subtyping results of the near full-length genome sequences.

No.	Accession Number	Genome Sequence Range ^1^	Rega 3.0	Comet	jpHMM	SimPlot	Final Result
11	MN736709	497–7842	A (A1)	A1	A1, K, A1	A1/K or 45_cpx	A|A/U|A|K|A
12	MN736698	497–9496	B	B	B	B	B
25	MN736699	497–9487	A (A1)	A1	A1	A6	A6
29	MN736700	497–9496	B	B	B	B	B
31	MN736710	497–7843	C	unassigned; 60_BC, B, 82_cpx, 01_AE, 58_01B, 01_AE, 58_01B, 01_AE, 58_01B, 59_01B, 58_01B, 59_01B, 58_01B, 59_01B, 01_AE, 59_01B, 58_01B, 02_AG, 58_01B, 02_AG, A1, 69_01B, 59_01B, 69_01B, 53_01B, 69_01B, 53_01B, 69_01B, 55_01[..]	C, B, C, 01_AE, C	60_BC	60_BC|01_AE|60_BC
34	MN736701	497–9496	recombinant of 13_cpx, G, A1, J, H	unassigned; G, A1, 01_AE, D, J, C, A2, C, B, D, B, D, B, D, J, C, A1, 01_AE	A1, G, D, J, B, J, G, A1, 01_AE	complex	U|A|G|U|G|J| G|U|J|U|G| A|01_AE
37	MN736702	497–9433	recombinant of 02_AG, B, G, A1	unassigned; 02_AG, 20_BG, 56_cpx, 20_BG, 56_cpx, 90_BF1, B, 56_cpx, B, 56_cpx, B, 71_BF1, 51_01B, 71_BF1, 39_BF, B, 69_01B, B, 69_01B, B, 69_01B, B, 56_cpx, 19_cpx, 24_BG, 19_cpx, 56_cpx, 24_BG, 56_cpx, G, 14_BG, 43_02G, G, 43_02G[..]	A1, B, A1, G, B, A1, G, B, A1, B, G	56_cpx, B	56_cpx|B|56_cpx
39	MN736703	497–9496	01_AE	unassigned; 01_AE, B, D	01_AE	01_AE	01_AE
43	MN736704	497–9412	B	unassigned; B, C, G	B	B, B/D, B, B/D	B
54	MN736705	497–9445	02_AG	02_AG	A1, G, A1, G, A1, G, A1, G, A1	02_AG	02_AG|A|02_AG
56	MN736706	497–9496	B	B	B	B	B
65	MN736707	497–9496	B	B	B	B	B
68	MN736708	497–9496	recombinant of G, A1, D, B, F1	unassigned; G, D, B, D, B, D, A1	A1, G, D, B, A1, G, A2, G, A1, G, A1, G	complex	U|19_cpx|G|19_cpx| B|19_cpx|A|19_cpx|U|19_cpx|G|19_cpx|G|19_cpx|G

^1^ nucleotide position according to HXB2, U = unknown.

## Supplementary Materials

The following are available online at https://www.mdpi.com/1999-4915/12/1/63/s1, Figure S1: SimPlot bootscan analysis of the Slovenian near full-length genome sequences determined to be non-recombinant, Figure S2: Phylogenetic analyses of the segments of the alignment of the near full-length genome sequences determined to be recombinant.
