# Supplementary material for: HIV-1 Unique Recombinant Forms Identified in Slovenia and Their Characterization by Near Full-Length Genome Sequencing
Source: Viruses. 2020 Jan 3;12(1):63. doi: 10.3390/v12010063 (PMC7019782; doi:10.3390/v12010063)

Supplementary Materials:

Figure S1: SimPlot bootscan analysis of the Slovenian near full-length genome sequences determined to be non-recombinant:

Sample 12

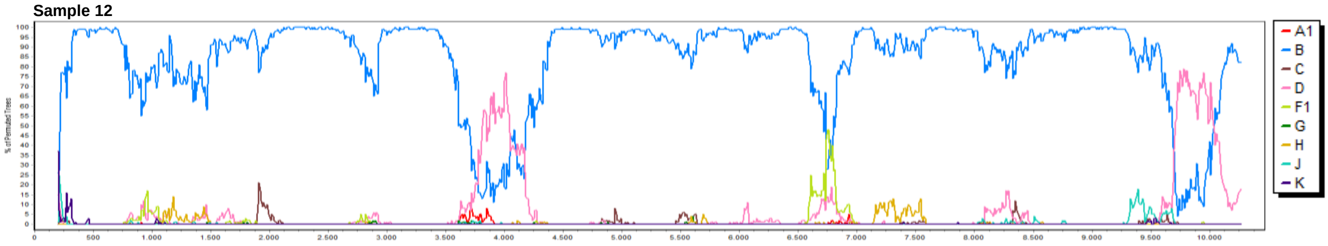

Sample 25

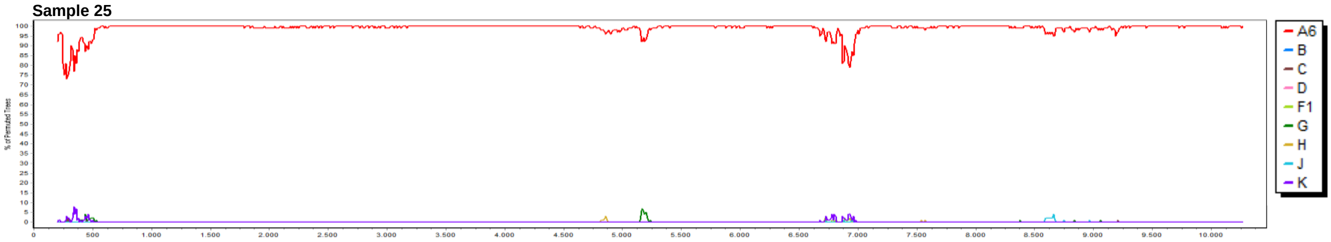

Sample 29

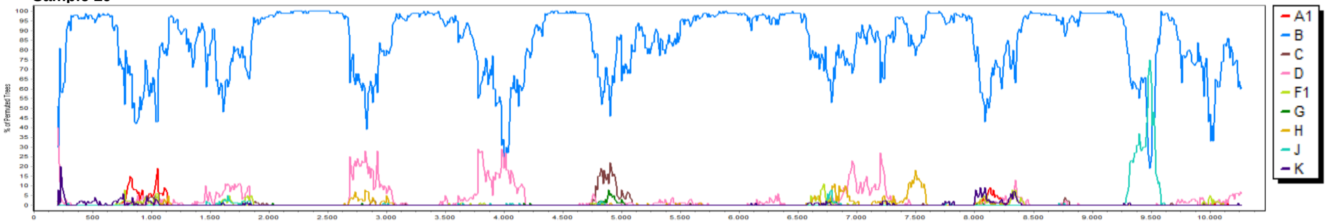

Sample 39

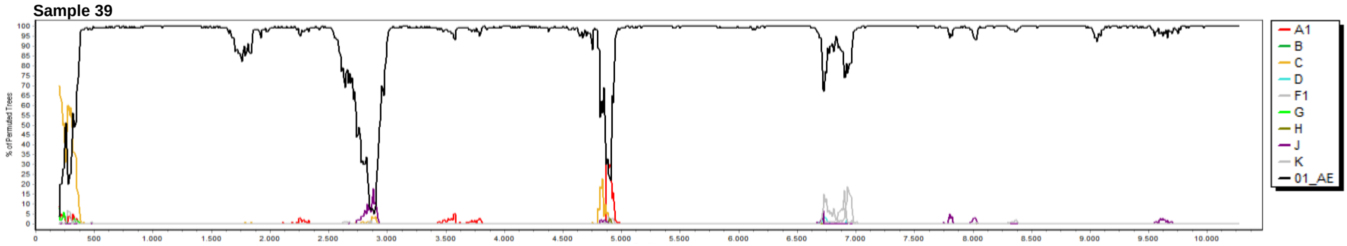

Sample 43

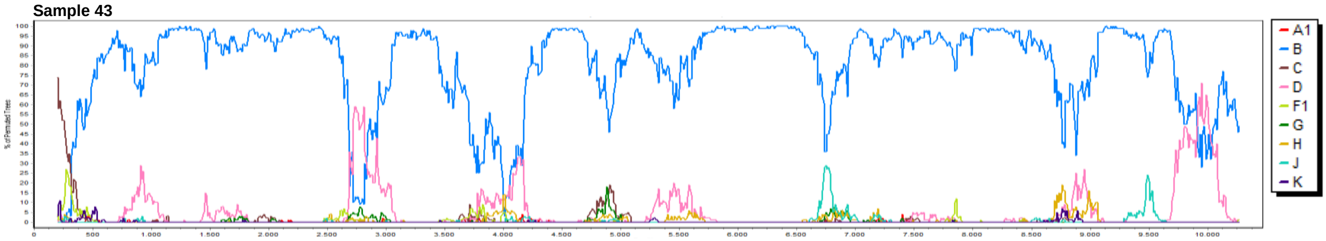

Sample 56

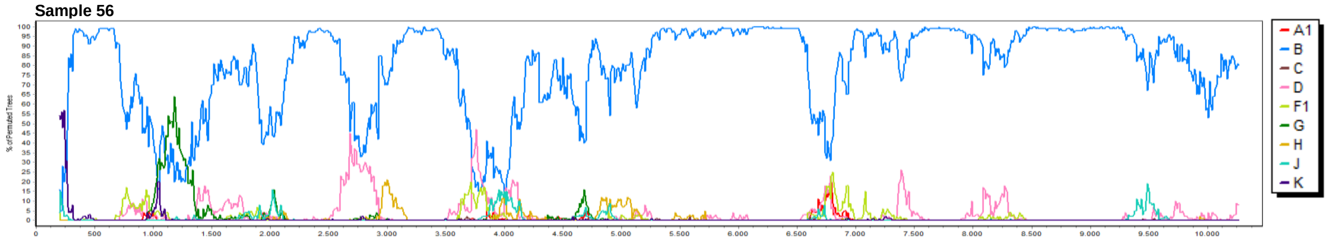

Sample 65

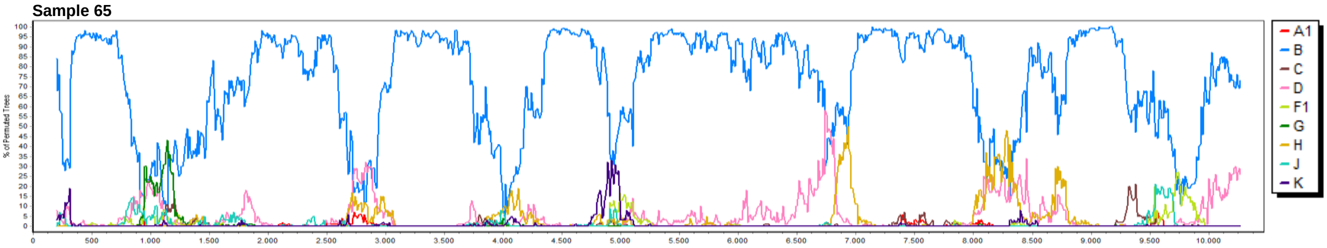

Figure S2: Phylogenetic analyses of the segments of the alignment of the near full-length genome sequences determined to be recombinant. Branches of major subtypes and CRFs were collapsed, for better visibility:

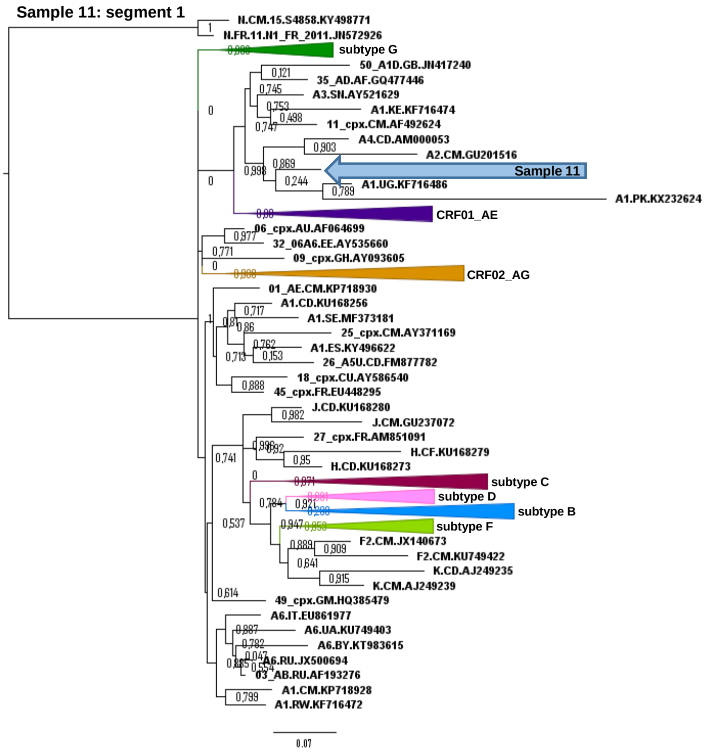

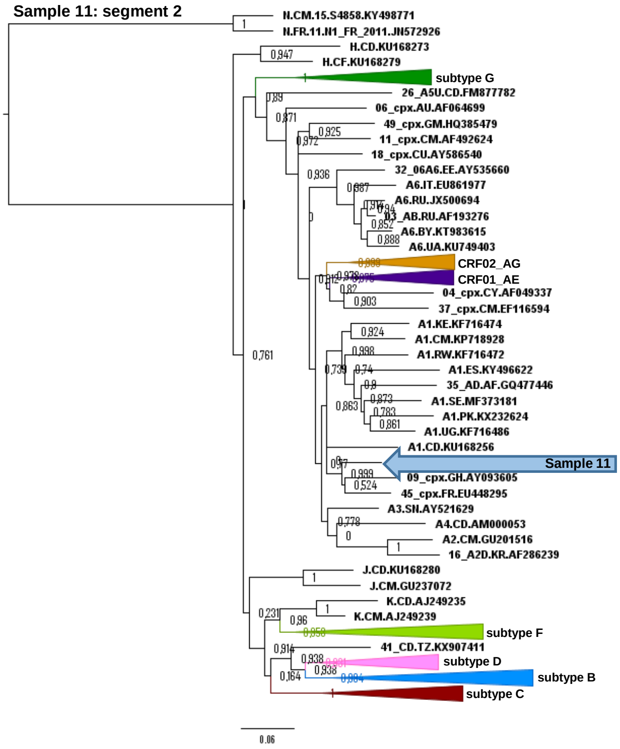

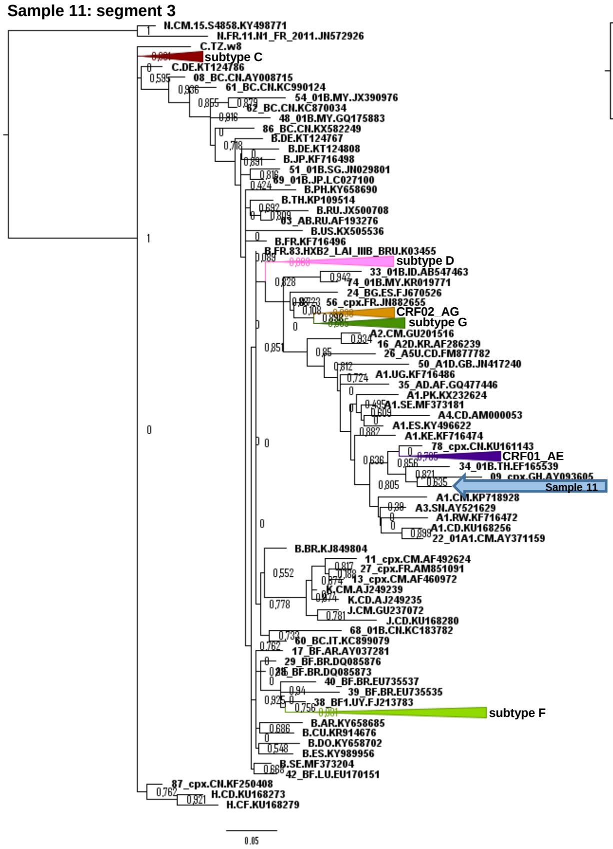

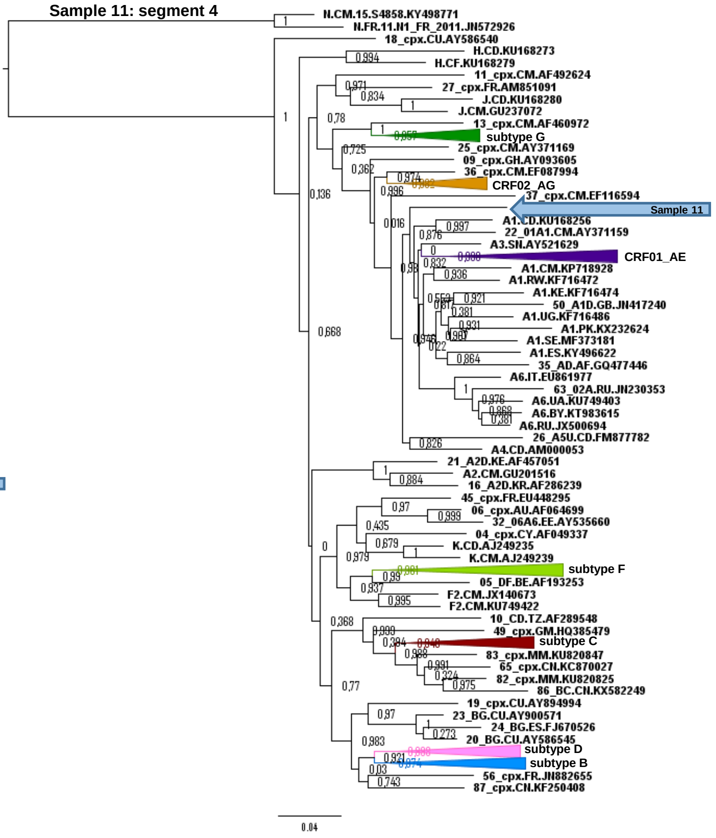

Sample 11: segment 5

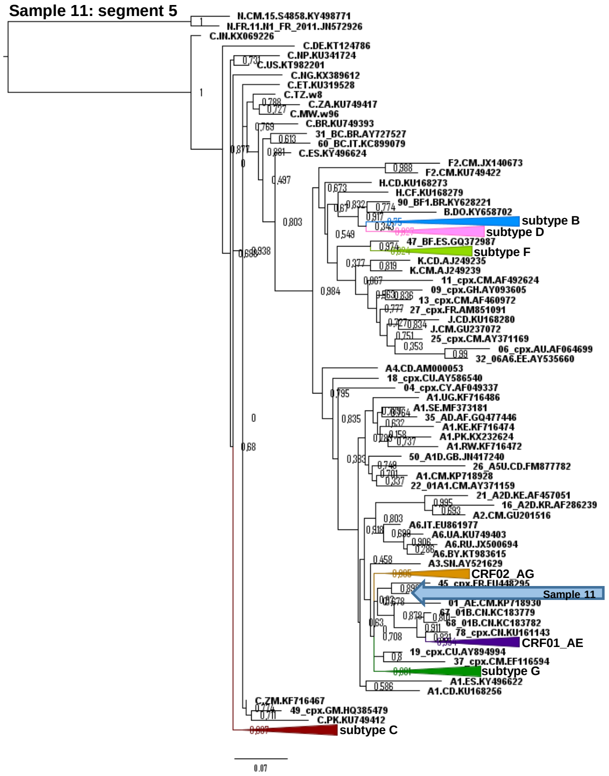

Sample 11: segment 6

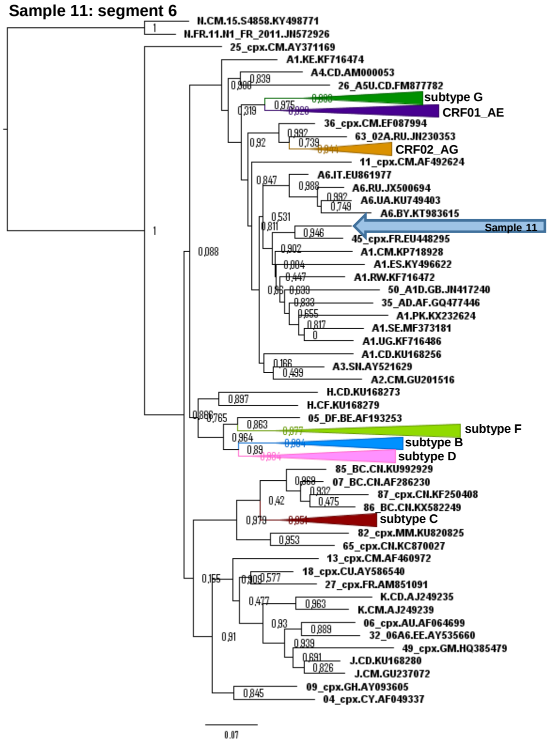

Sample 11: segment 7

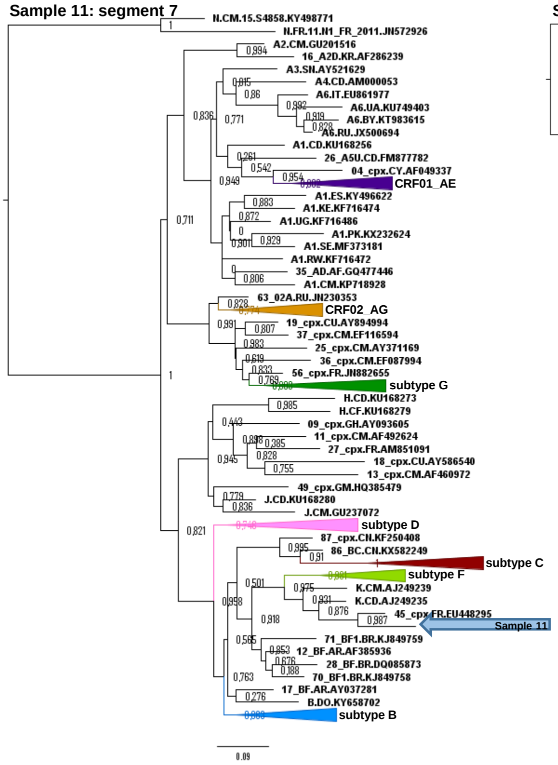

Sample 11: segment 8

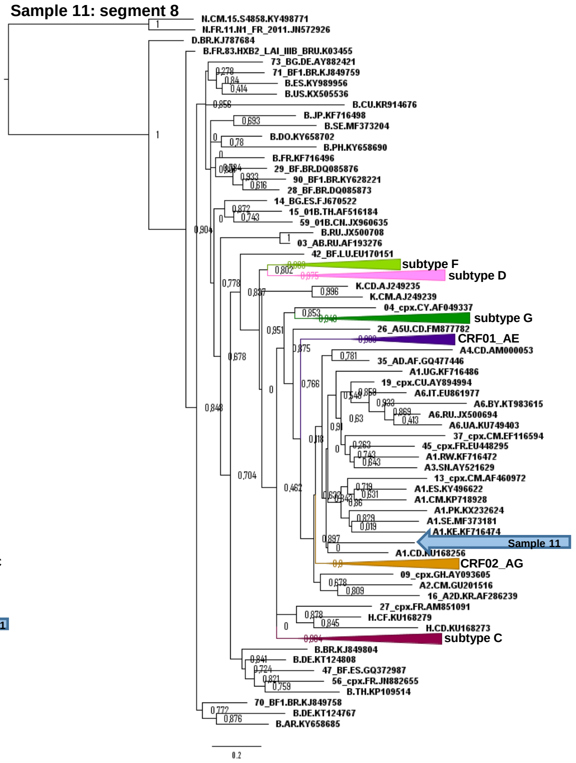

Sample 11: segment 9

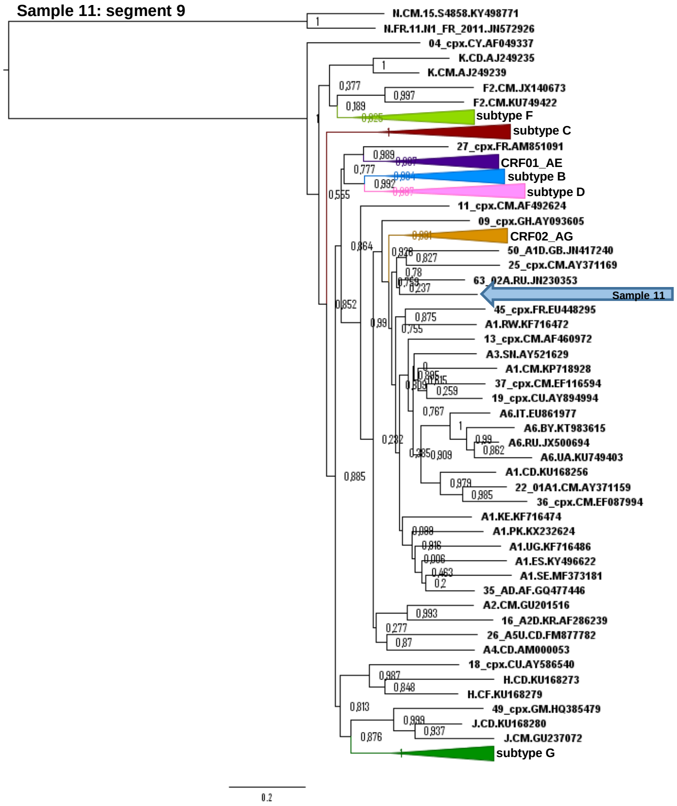

Sample 31: segment 1

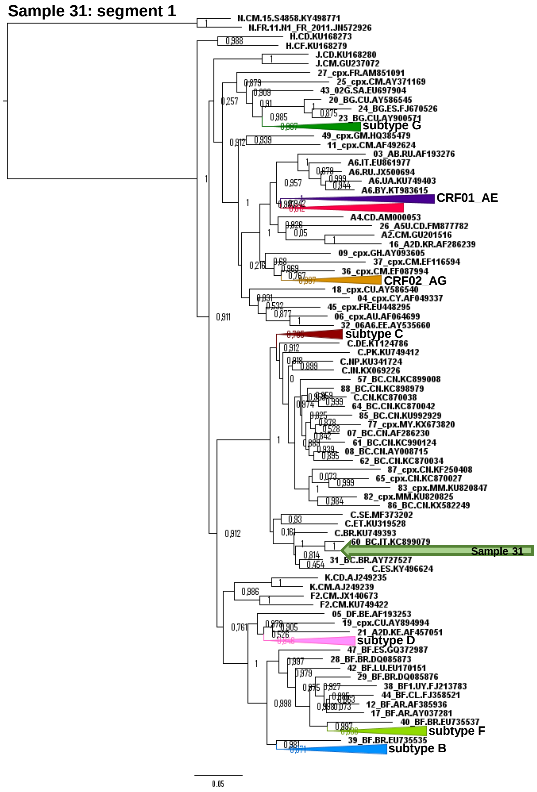

Sample 31: segment 2

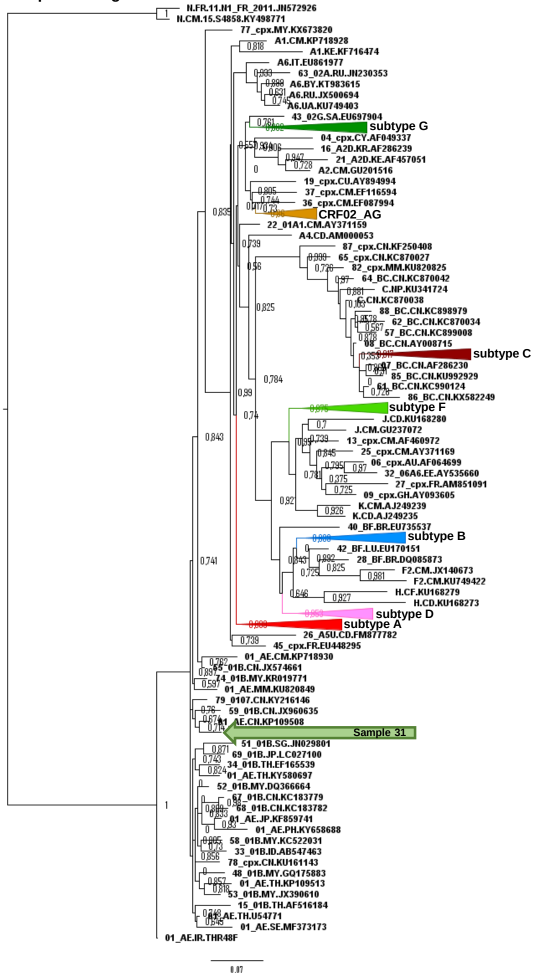

Sample 31: segment 3

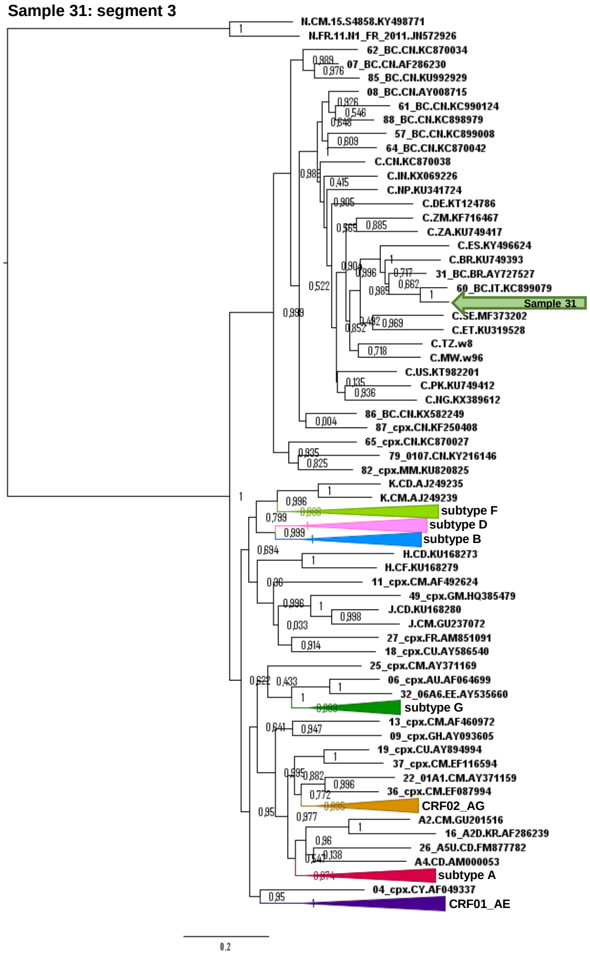

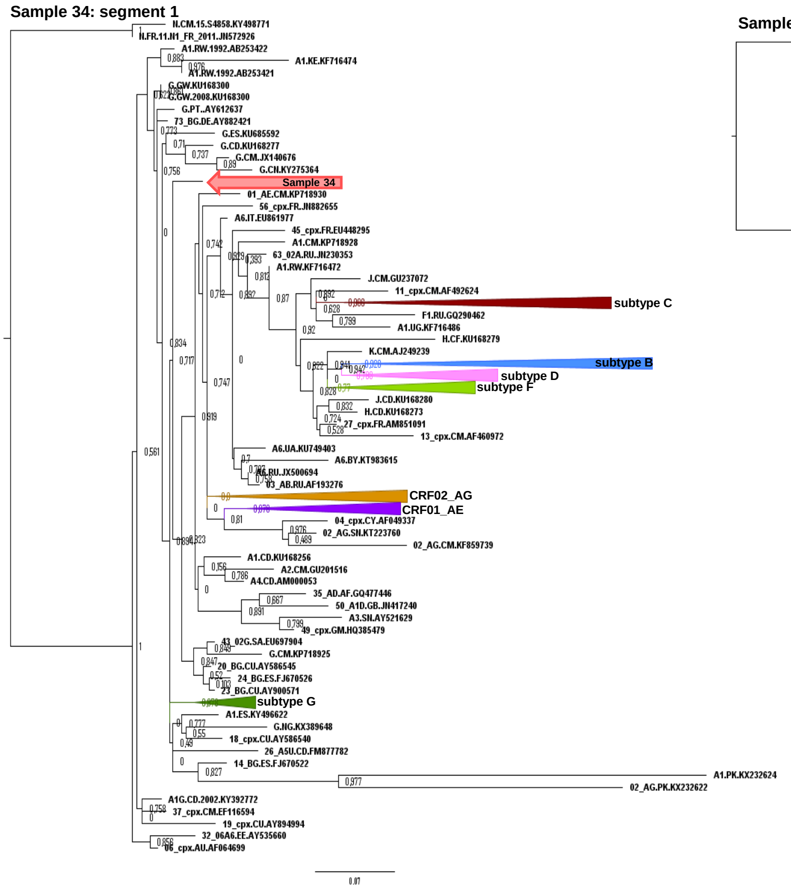
Sample 34: segment 1
Sample 34
subtype C
subtype B
subtype D
subtype F
CRF02_AG
CRF01_AE
subtype G

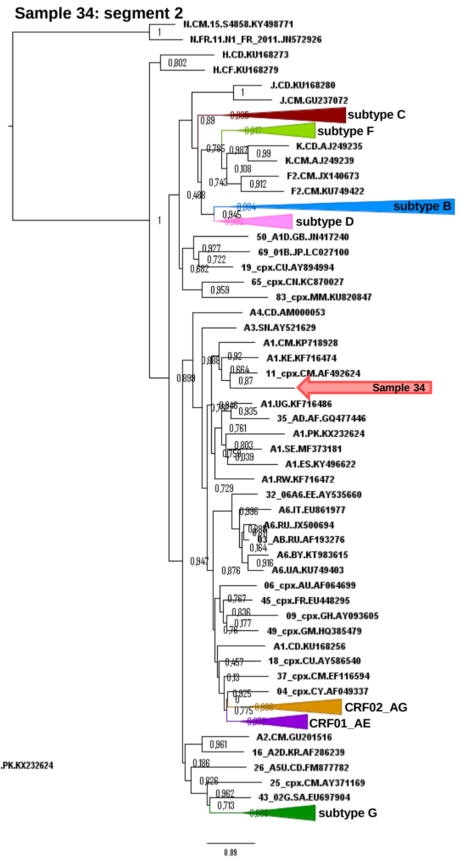
Sample 34: segment 2
subtype C
subtype F
subtype B
subtype D
Sample 34
CRF02_AG
CRF01_AE
subtype G

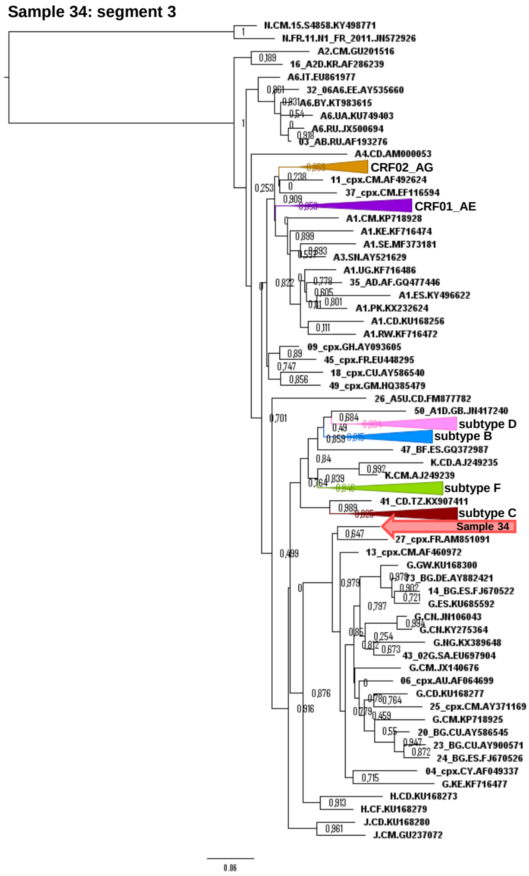
Sample 34: segment 3
CRF02_AG
CRF01_AE
subtype D
subtype B
subtype F
subtype C
Sample 34

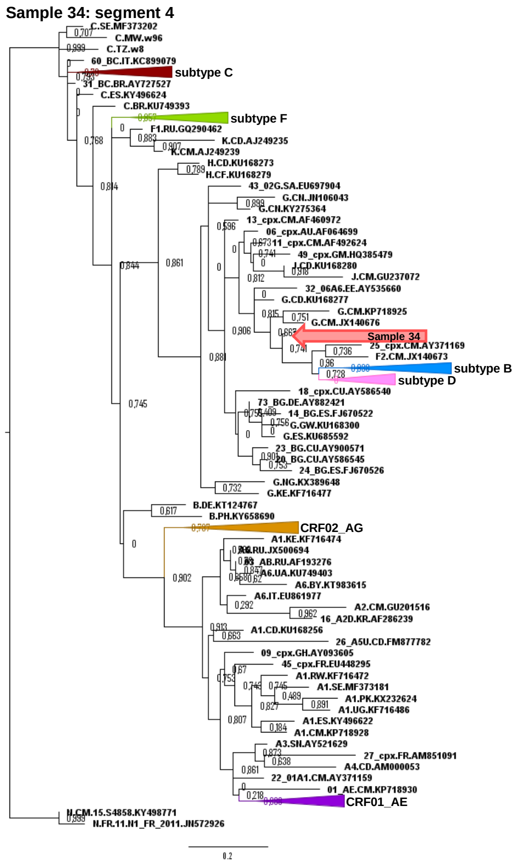
Sample 34: segment 4
subtype C
subtype F
Sample 34
subtype B
subtype D
CRF02_AG
CRF01_AE

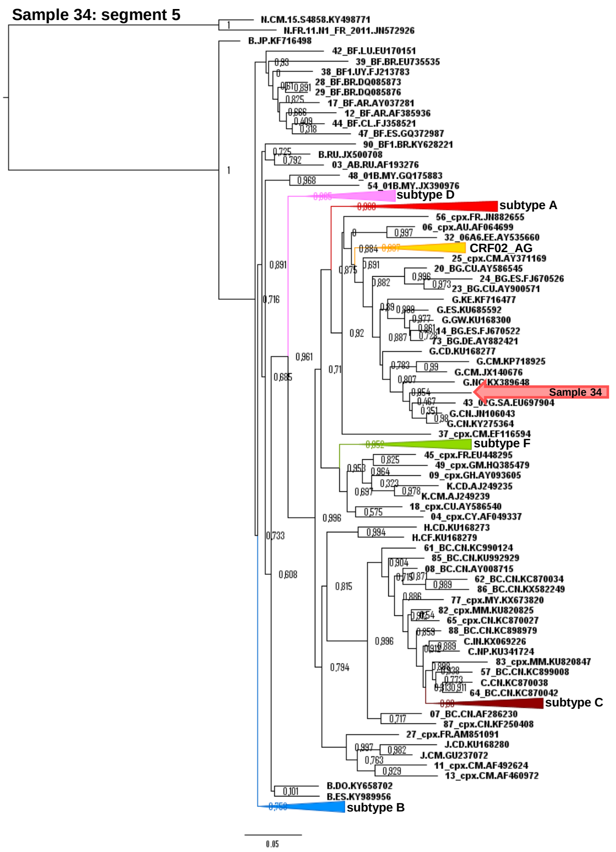
Sample 34: segment 5
subtype D
subtype A
CRF02_AG
Sample 34
subtype F
subtype C
subtype B
0.05

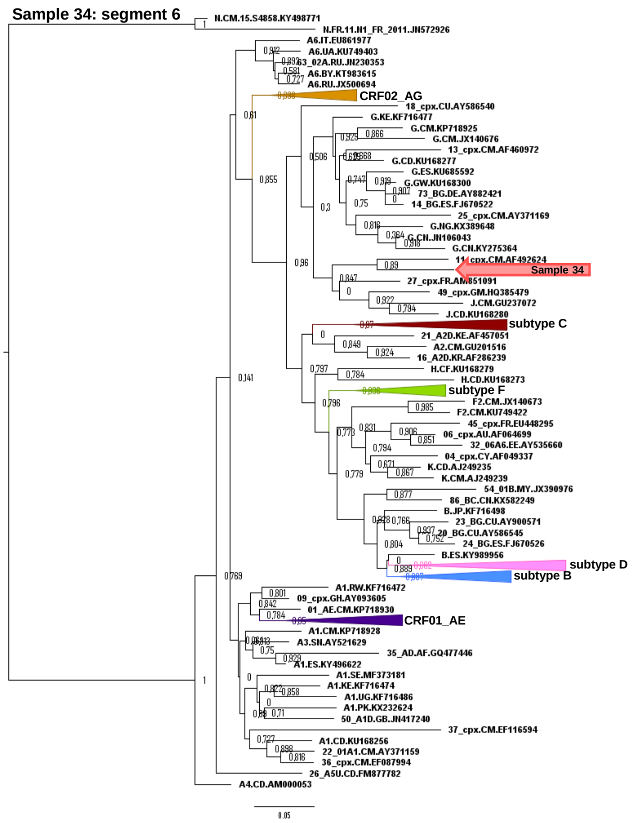
Sample 34: segment 6
CRF02_AG
Sample 34
subtype C
subtype F
subtype D
subtype B
CRF01_AE
0.05

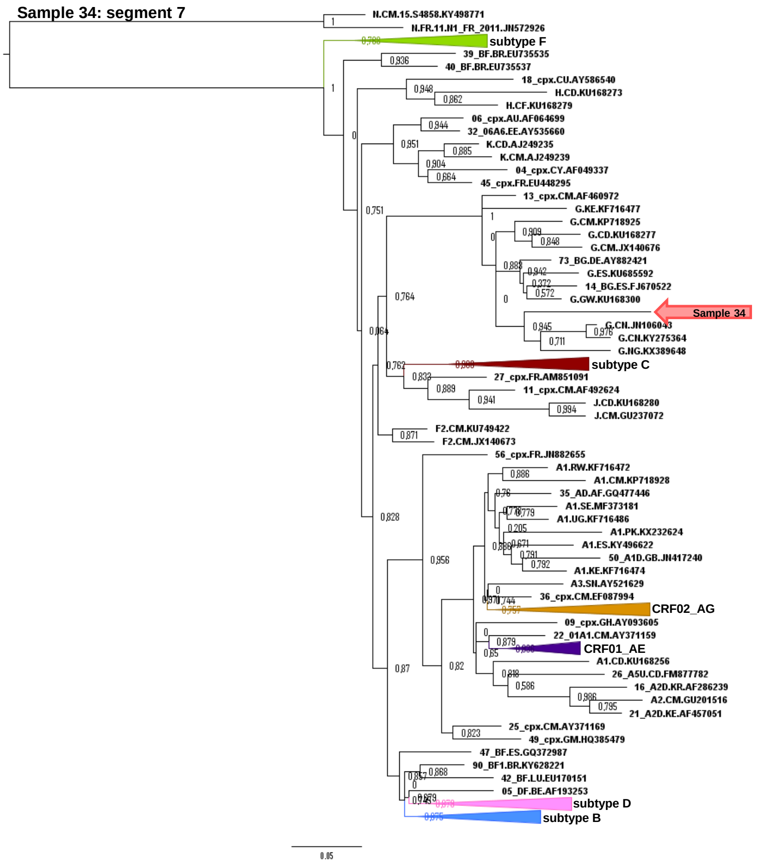
Sample 34: segment 7
subtype F
Sample 34
subtype C
CRF02_AG
CRF01_AE
subtype D
subtype B
0.05

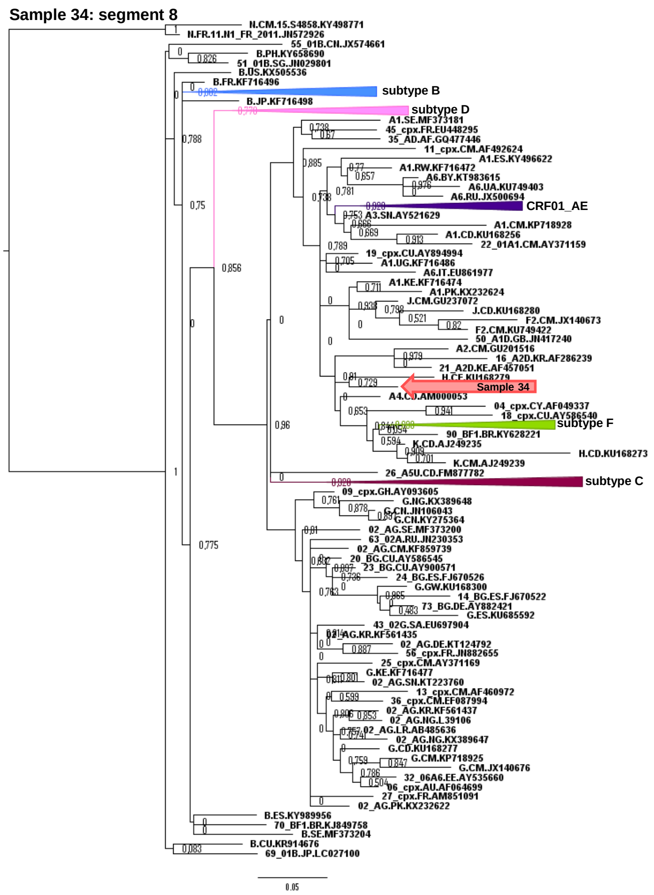
Sample 34: segment 8
subtype B
subtype D
CRF01_AE
Sample 34
subtype F
subtype C
0.05

Sample 34: segment 9

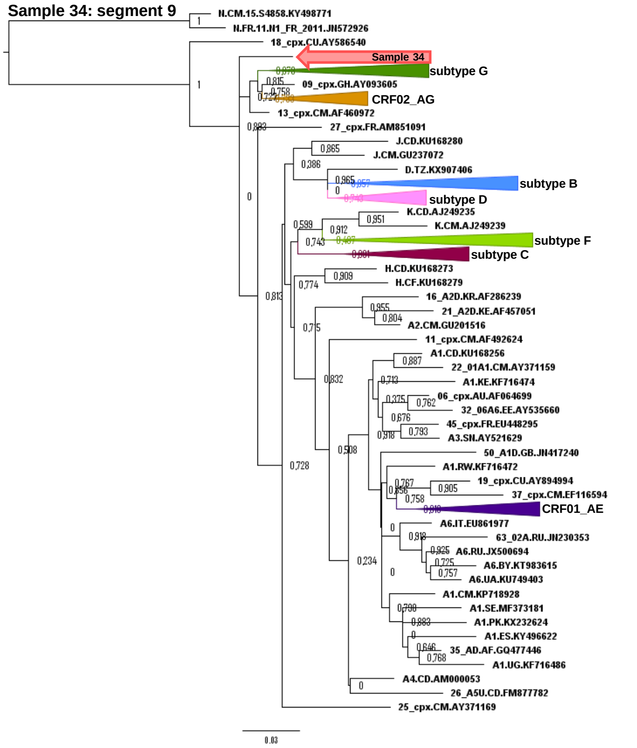

Sample 34: segment 10

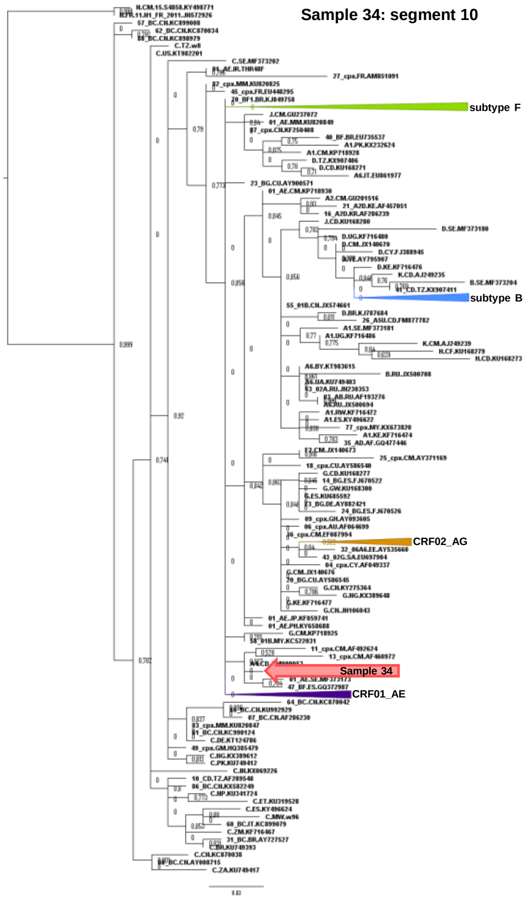

Sample 34: segment 11

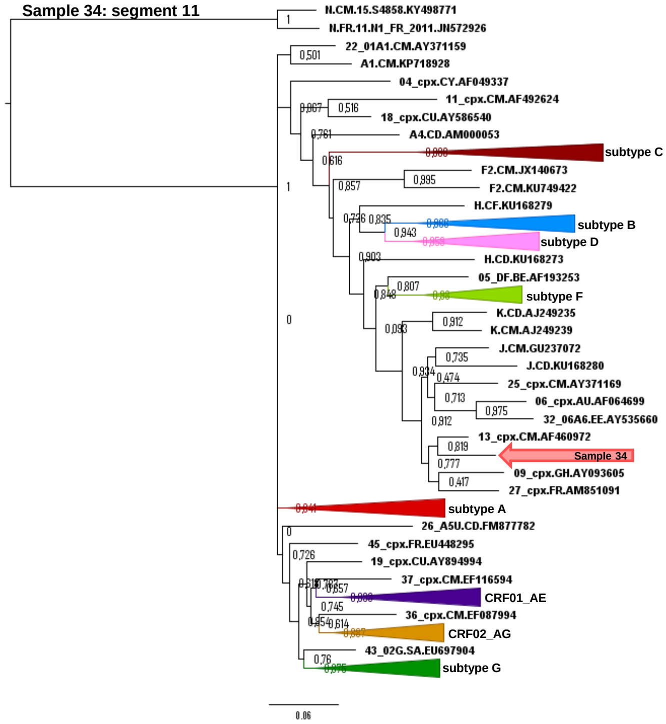

Sample 34: segment 12

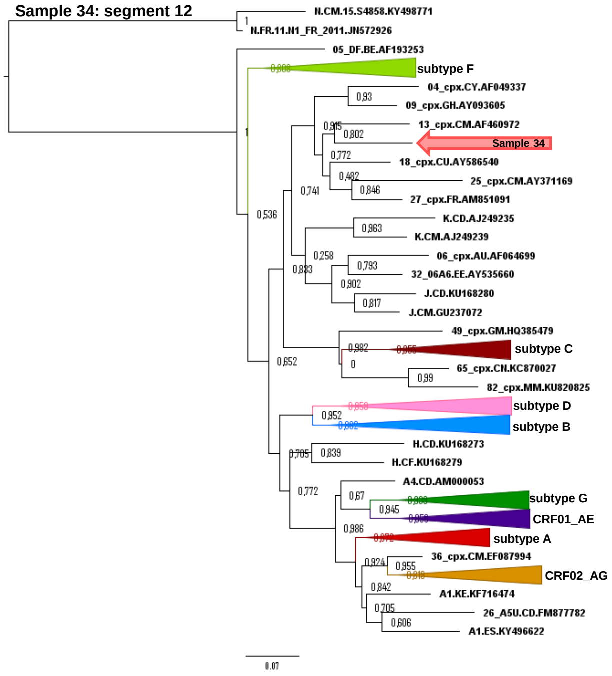

Sample 34: segment 13

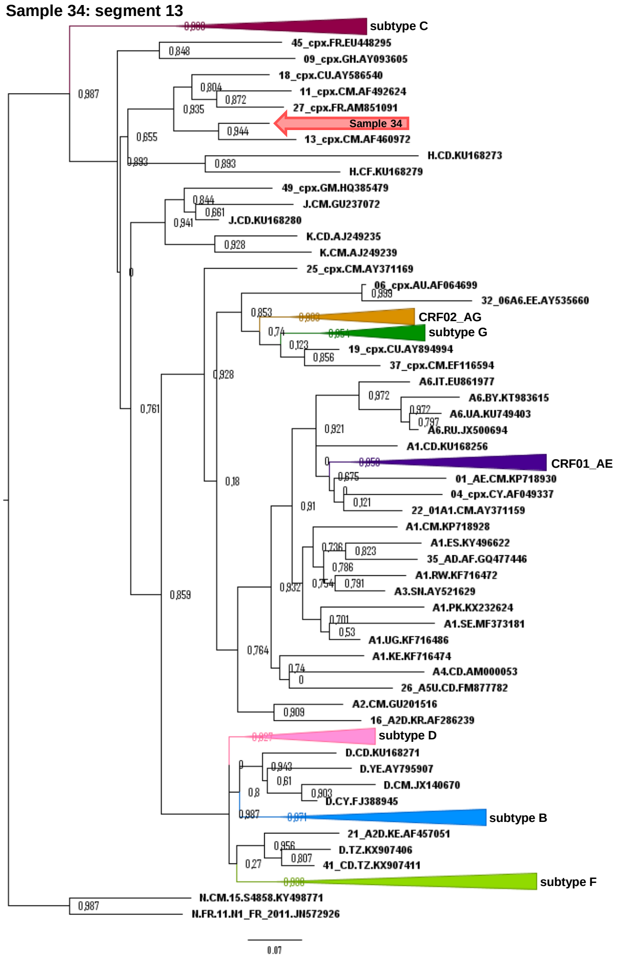

Sample 34: segment 14

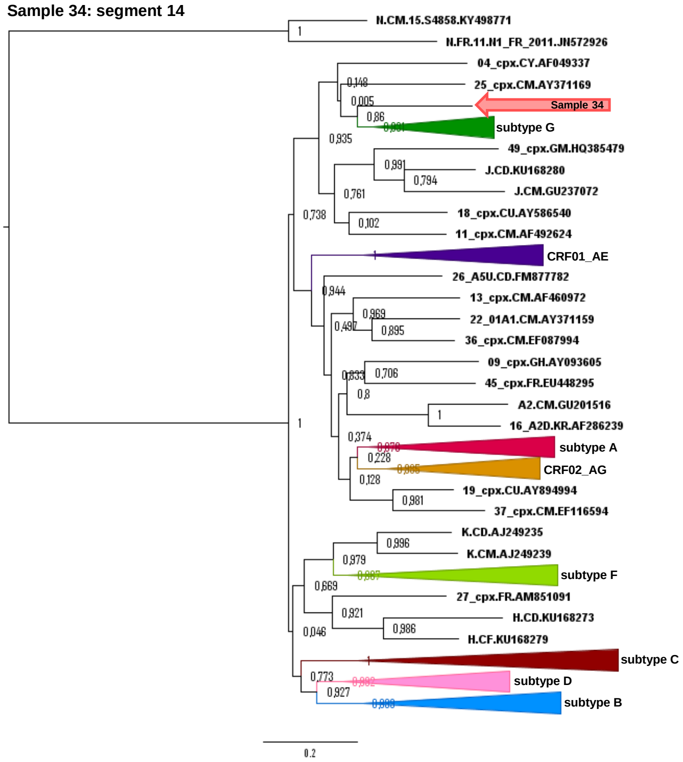

Sample 34: segment 15

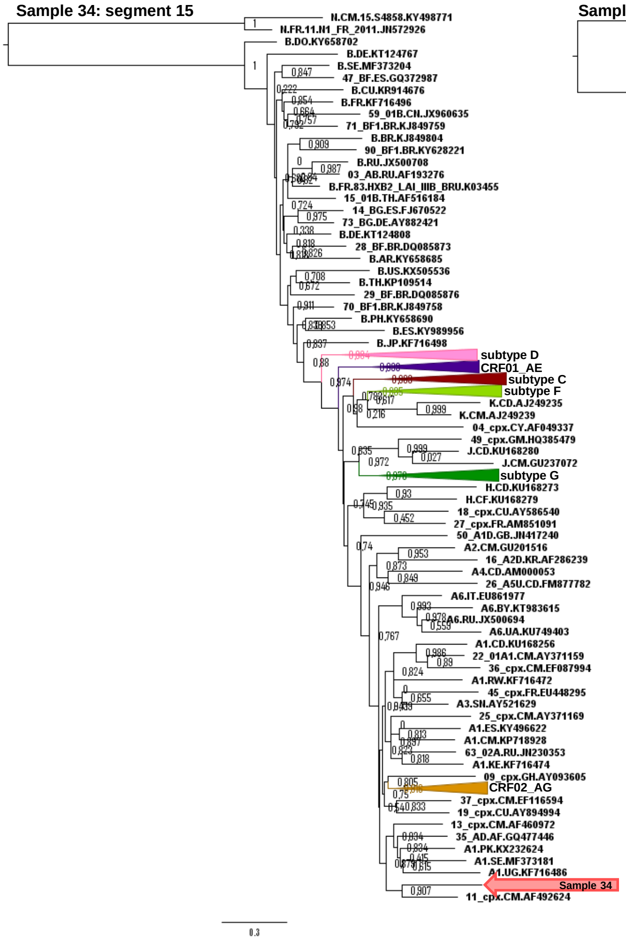

Sample 34: segment 16

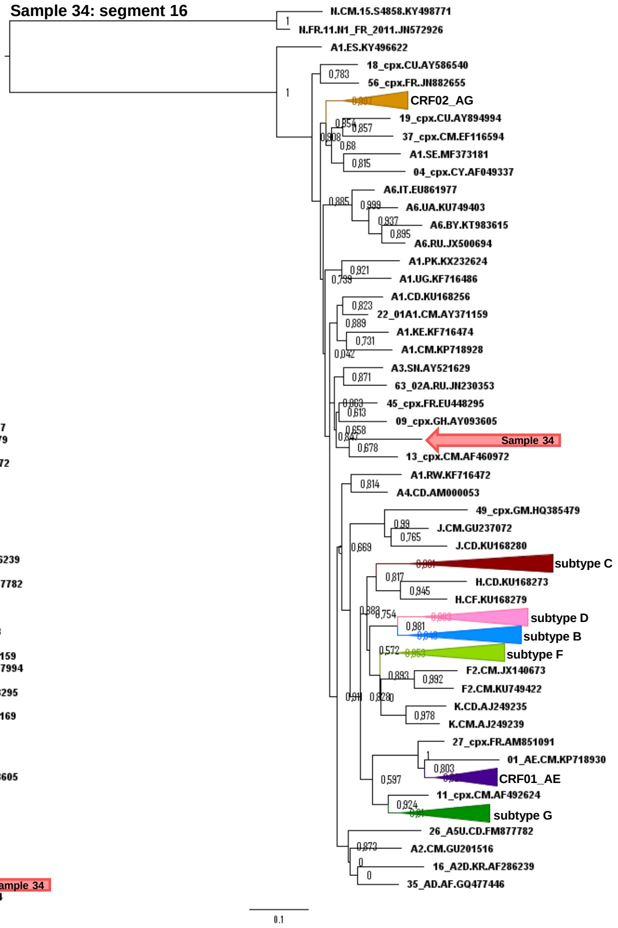

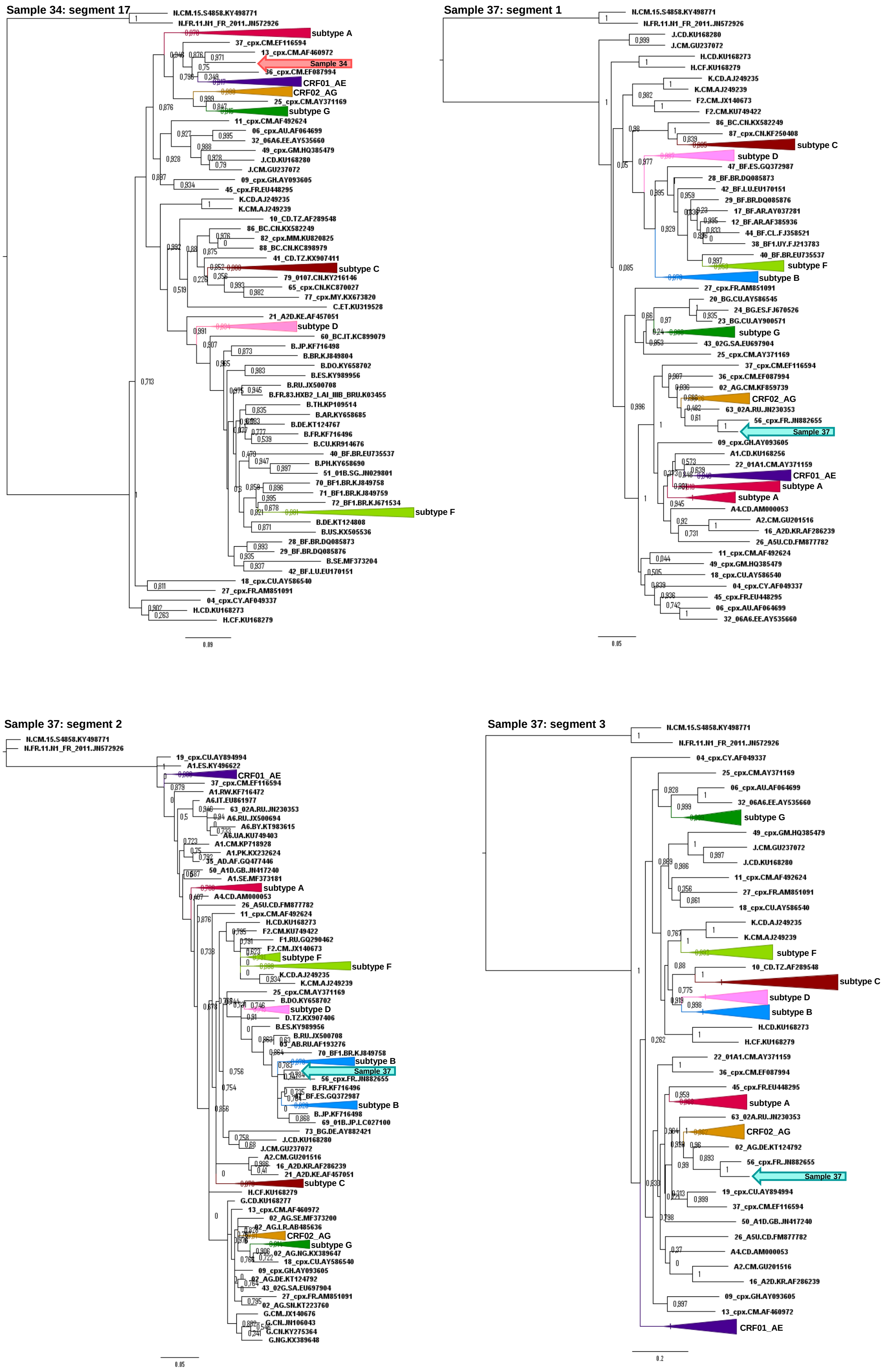
Sample 34: segment 17
subtype A
Sample 34
CRF01_AE
CRF02_AG
subtype G
subtype C
subtype D
subtype F
Sample 37: segment 1
subtype C
subtype D
subtype F
subtype B
subtype G
CRF02_AG
Sample 37
CRF01_AE
subtype A
Sample 37: segment 2
CRF01_AE
subtype A
subtype F
subtype D
subtype B
Sample 37
subtype C
CRF02_AG
subtype G
Sample 37: segment 3
subtype G
subtype F
subtype C
subtype D
subtype B
subtype A
CRF02_AG
Sample 37
CRF01_AE

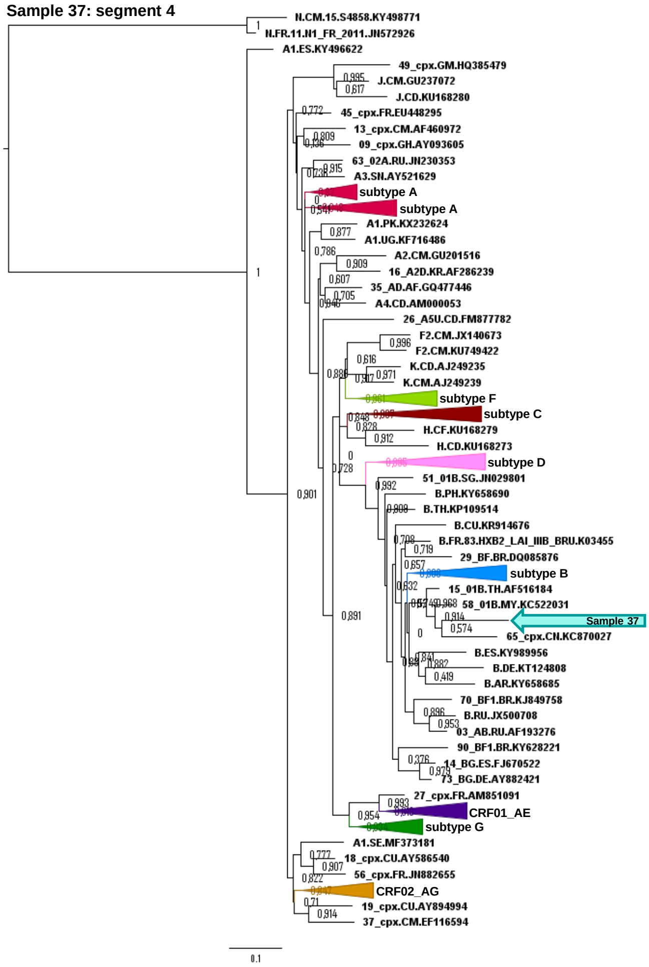

Sample 37: segment 4
subtype A
subtype A
subtype F
subtype C
subtype D
subtype B
Sample 37
CRF01_AE
subtype G
CRF02_AG
0.1

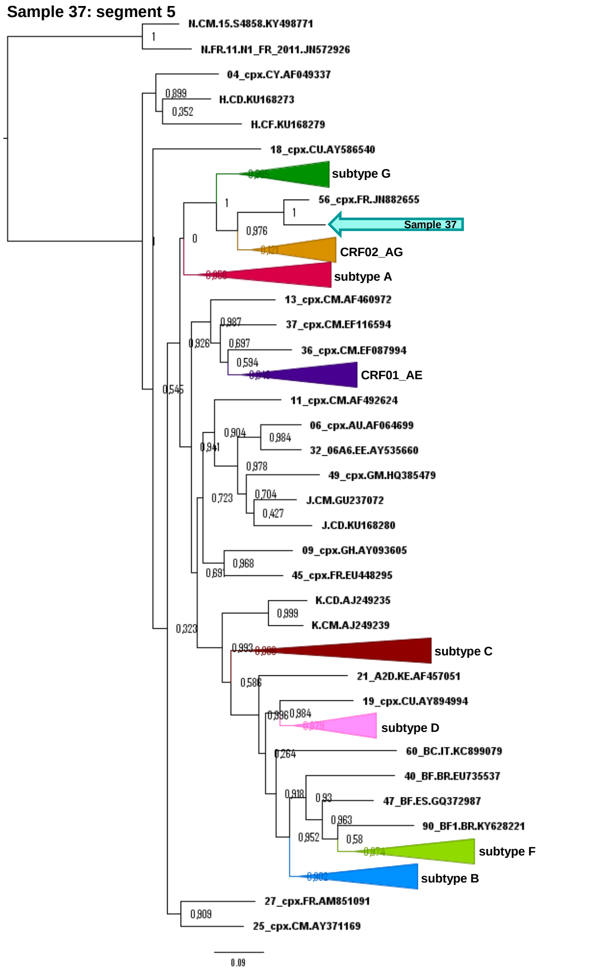

Sample 37: segment 5
subtype G
Sample 37
CRF02_AG
subtype A
CRF01_AE
subtype C
subtype D
subtype F
subtype B
0.09

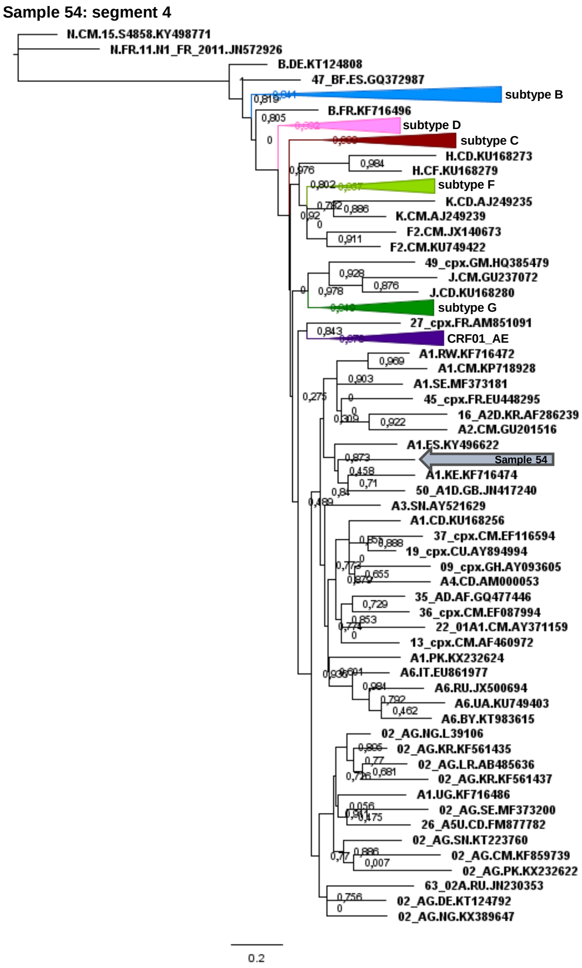

Sample 54: segment 4
subtype B
subtype D
subtype C
subtype F
subtype G
CRF01_AE
Sample 54
0.2

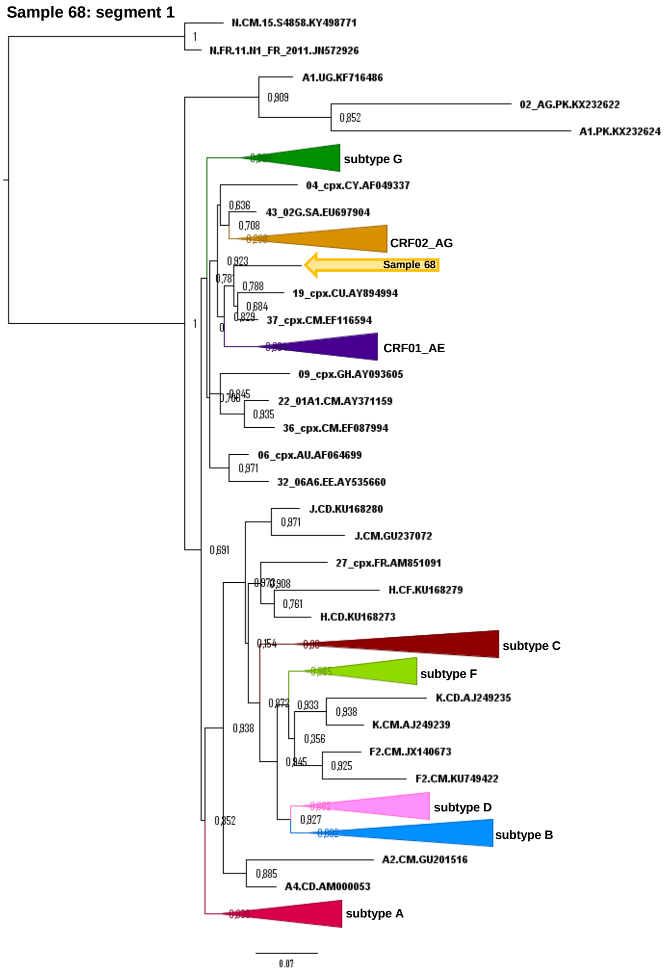

Sample 68: segment 1
subtype G
CRF02_AG
Sample 68
CRF01_AE
subtype C
subtype F
subtype D
subtype B
subtype A
0.07

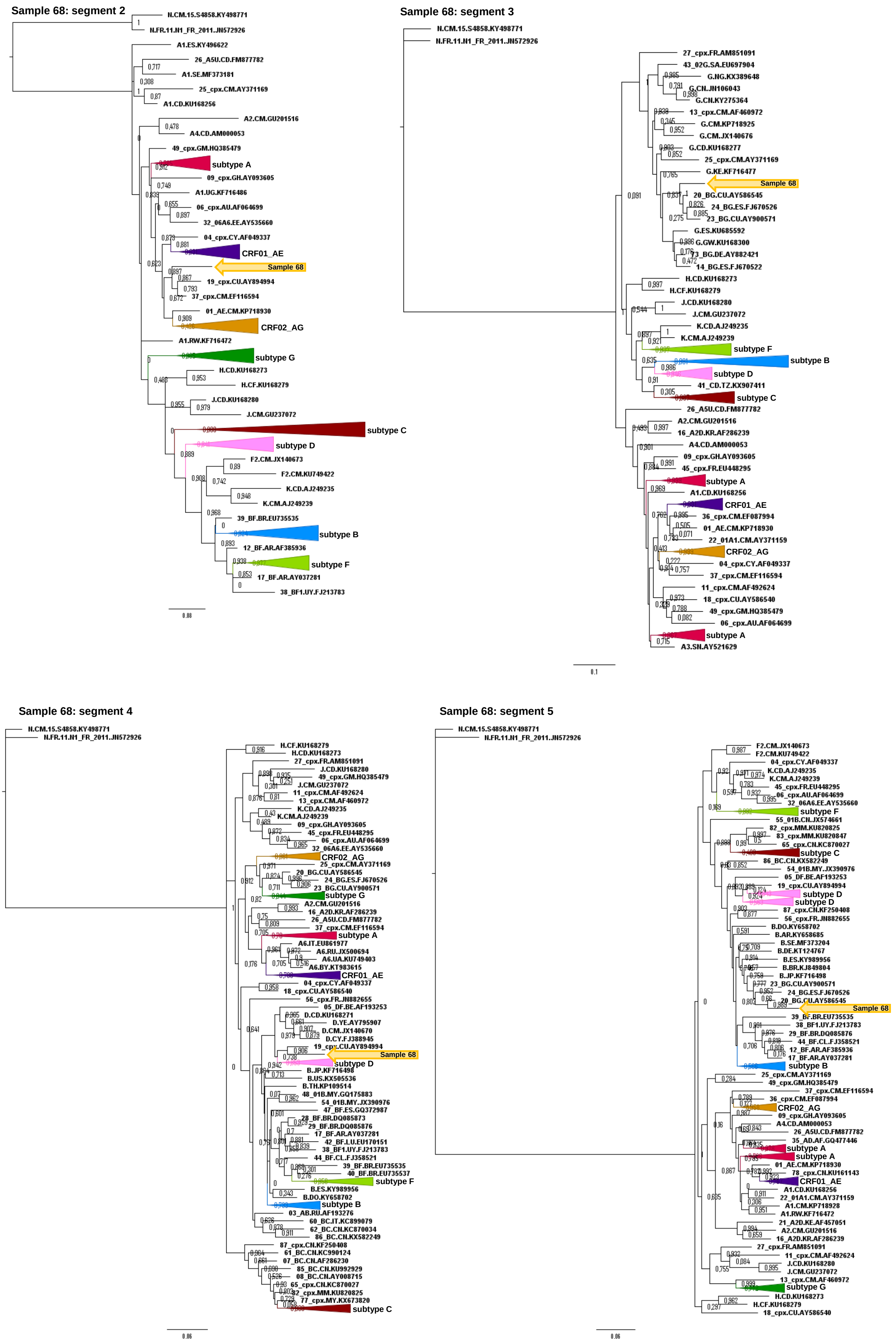

Sample 68: segment 2
N.CM.15.S4858.KY498771
N.FR.11.N1_FR_2011.JN572926
A1.ES.KY496622
26_A5U.CD.FM877782
A1.SE.MF373181
25_cpx.CM.AY371169
A1.CD.KU168256
A2.CM.GU201516
A4.CD.AM000053
49_cpx.GM.HQ385479
subtype A
09_cpx.GH.AY093605
A1.UG.KF716486
06_cpx.AU.AF064699
32_06A6.EE.AY535660
04_cpx.CY.AF049337
CRF01_AE
Sample 68
19_cpx.CU.AY894994
37_cpx.CM.EF116594
01_AE.CM.KP718930
CRF02_AG
A1.RW.KF716472
subtype G
H.CD.KU168273
H.CF.KU168279
J.CD.KU168280
J.CM.GU237072
subtype C
subtype D
F2.CM.JX140673
F2.CM.KU749422
K.CD.AJ249235
K.CM.AJ249239
39_BF.BR.EU735535
subtype B
12_BF.AR.AF385936
subtype F
17_BF.AR.AY037281
38_BF1.UY.FJ213783
0.08
Sample 68: segment 3
N.CM.15.S4858.KY498771
N.FR.11.N1_FR_2011.JN572926
27_cpx.FR.AM851091
43_02G.SA.EU697904
G.NG.KX389648
G.CN.JN106043
G.CN.KY275364
13_cpx.CM.AF460972
G.CM.KP718925
G.CM.JX140676
G.CD.KU168277
25_cpx.CM.AY371169
G.KE.KF716477
Sample 68
20_BG.CU.AY586545
24_BG.ES.FJ670526
23_BG.CU.AY900571
G.ES.KU685592
G.GW.KU168300
73_BG.DE.AY882421
14_BG.ES.FJ670522
H.CD.KU168273
H.CF.KU168279
J.CD.KU168280
J.CM.GU237072
K.CD.AJ249235
K.CM.AJ249239
subtype F
subtype B
subtype D
41_CD.TZ.KX907411
subtype C
26_A5U.CD.FM877782
A2.CM.GU201516
16_A2D.KR.AF286239
A4.CD.AM000053
09_cpx.GH.AY093605
45_cpx.FR.EU448295
subtype A
A1.CD.KU168256
CRF01_AE
36_cpx.CM.EF087994
01_AE.CM.KP718930
22_01A1.CM.AY371159
CRF02_AG
04_cpx.CY.AF049337
37_cpx.CM.EF116594
11_cpx.CM.AF492624
18_cpx.CU.AY586540
49_cpx.GM.HQ385479
06_cpx.AU.AF064699
subtype A
A3.SN.AY521629
0.1
Sample 68: segment 4
N.CM.15.S4858.KY498771
N.FR.11.N1_FR_2011.JN572926
H.CF.KU168279
H.CD.KU168273
27_cpx.FR.AM851091
J.CD.KU168280
49_cpx.GM.HQ385479
J.CM.GU237072
11_cpx.CM.AF492624
13_cpx.CM.AF460972
K.CD.AJ249235
K.CM.AJ249239
09_cpx.GH.AY093605
45_cpx.FR.EU448295
06_cpx.AU.AF064699
32_06A6.EE.AY535660
CRF02_AG
25_cpx.CM.AY371169
20_BG.CU.AY586545
24_BG.ES.FJ670526
23_BG.CU.AY900571
subtype G
A2.CM.GU201516
16_A2D.KR.AF286239
26_A5U.CD.FM877782
37_cpx.CM.EF116594
subtype A
A6.IT.EU861977
A6.RU.JX500694
A6.UA.KU749403
A6.BY.KT983615
CRF01_AE
04_cpx.CY.AF049337
18_cpx.CU.AY586540
56_cpx.FR.JN882655
05_DF.BE.AF193253
D.CD.KU168271
D.YE.AY795907
D.CM.JX140670
D.CY.FJ388945
19_cpx.CU.AY894994
Sample 68
subtype D
B.JP.KF716498
B.US.KX505536
B.TH.KP109514
48_01B.MY.GQ175883
54_01B.MY.JX390976
47_BF.ES.GQ372987
28_BF.BR.DQ085873
29_BF.BR.DQ085876
17_BF.AR.AY037281
42_BF.LU.EU170151
38_BF1.UY.FJ213783
44_BF.CL.FJ358521
39_BF.BR.EU735535
40_BF.BR.EU735537
subtype F
B.ES.KY989956
B.DO.KY658702
subtype B
03_AB.RU.AF193276
60_BC.IT.KC899079
62_BC.CN.KC870034
86_BC.CN.KX582249
87_cpx.CN.KF250408
61_BC.CN.KC990124
07_BC.CN.AF286230
85_BC.CN.KU992929
08_BC.CN.AY008715
65_cpx.CN.KC870027
82_cpx.MM.KU820825
77_cpx.MY.KX673820
subtype C
0.06
Sample 68: segment 5
N.CM.15.S4858.KY498771
N.FR.11.N1_FR_2011.JN572926
F2.CM.JX140673
F2.CM.KU749422
04_cpx.CY.AF049337
K.CD.AJ249235
K.CM.AJ249239
45_cpx.FR.EU448295
06_cpx.AU.AF064699
32_06A6.EE.AY535660
subtype F
55_01B.CN.JX574661
82_cpx.MM.KU820825
83_cpx.MM.KU820847
65_cpx.CN.KC870027
subtype C
86_BC.CN.KX582249
54_01B.MY.JX390976
05_DF.BE.AF193253
19_cpx.CU.AY894994
subtype D
subtype D
87_cpx.CN.KF250408
56_cpx.FR.JN882655
B.DO.KY658702
B.AR.KY658685
B.SE.MF373204
B.DE.KT124767
B.ES.KY989956
B.BR.KJ849804
B.JP.KF716498
23_BG.CU.AY900571
24_BG.ES.FJ670526
20_BG.CU.AY586545
Sample 68
39_BF.BR.EU735535
38_BF1.UY.FJ213783
29_BF.BR.DQ085876
44_BF.CL.FJ358521
12_BF.AR.AF385936
17_BF.AR.AY037281
subtype B
25_cpx.CM.AY371169
49_cpx.GM.HQ385479
37_cpx.CM.EF116594
36_cpx.CM.EF087994
CRF02_AG
09_cpx.GH.AY093605
A4.CD.AM000053
26_A5U.CD.FM877782
35_AD.AF.GQ477446
subtype A
subtype A
01_AE.CM.KP718930
78_cpx.CN.KU161143
CRF01_AE
A1.CD.KU168256
22_01A1.CM.AY371159
A1.CM.KP718928
A1.RW.KF716472
21_A2D.KE.AF457051
A2.CM.GU201516
16_A2D.KR.AF286239
27_cpx.FR.AM851091
11_cpx.CM.AF492624
J.CD.KU168280
J.CM.GU237072
13_cpx.CM.AF460972
subtype G
H.CD.KU168273
H.CF.KU168279
18_cpx.CU.AY586540
0.06

Sample 68: segment 6

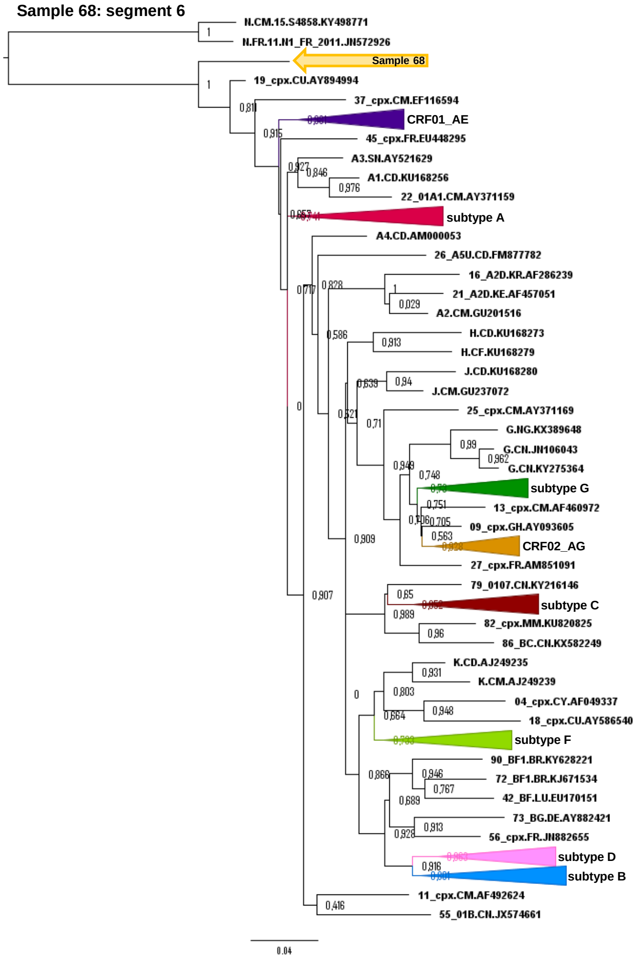

Sample 68: segment 7

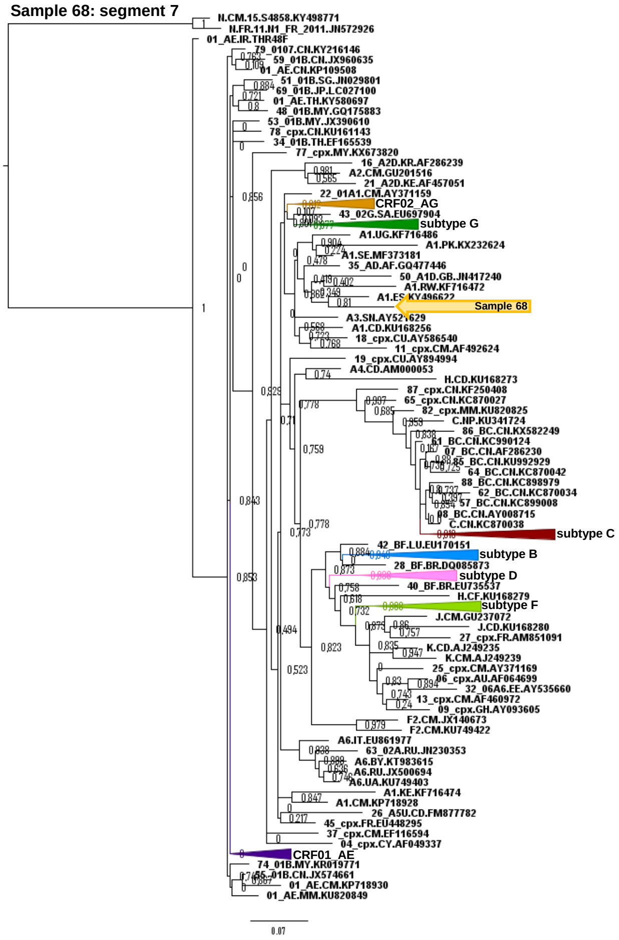

Sample 68: segment 8

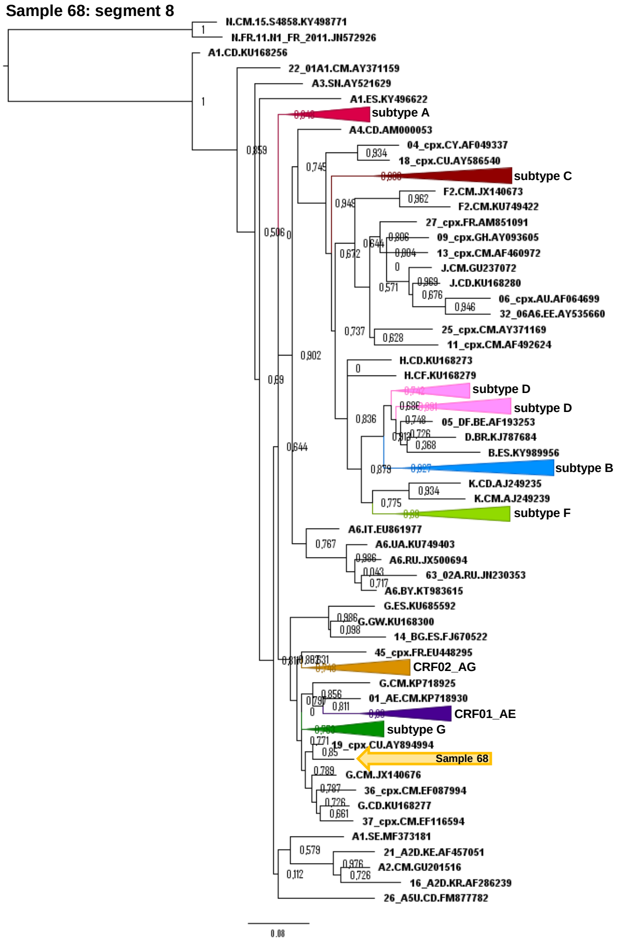

Sample 68: segment 9

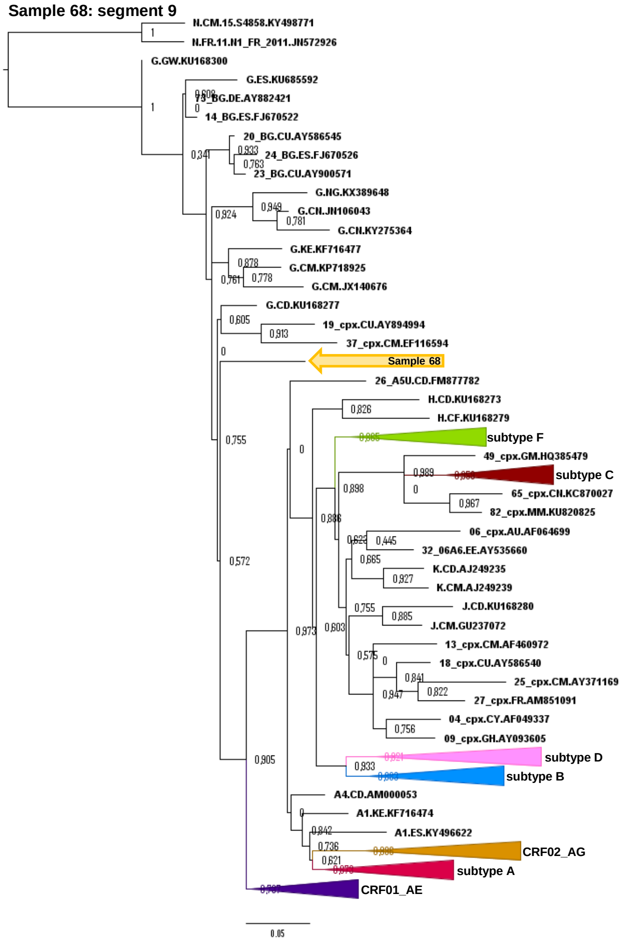

Sample 68: segment 10

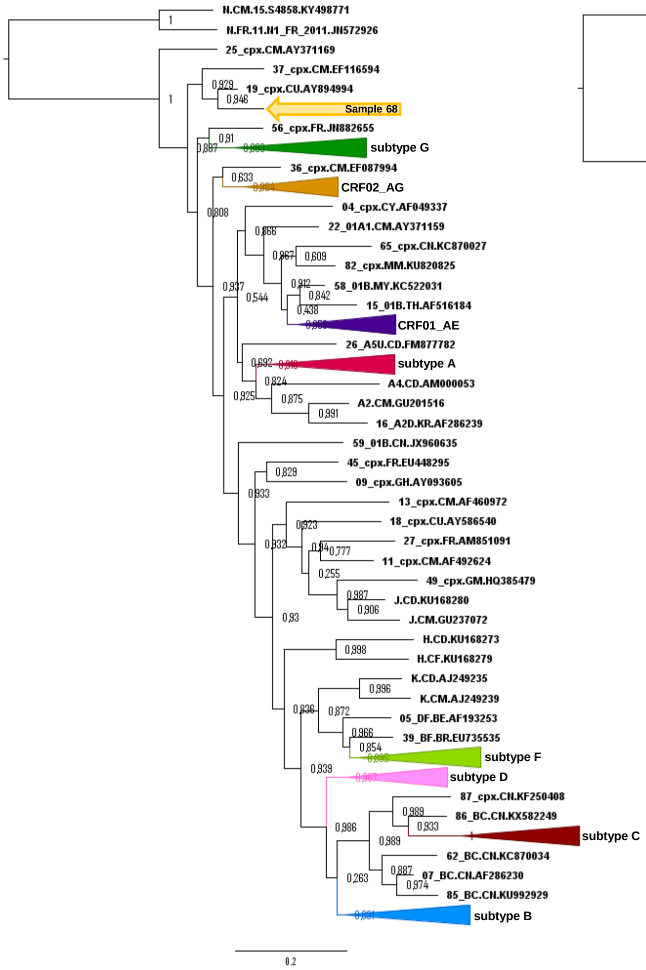

Sample 68: segment 11

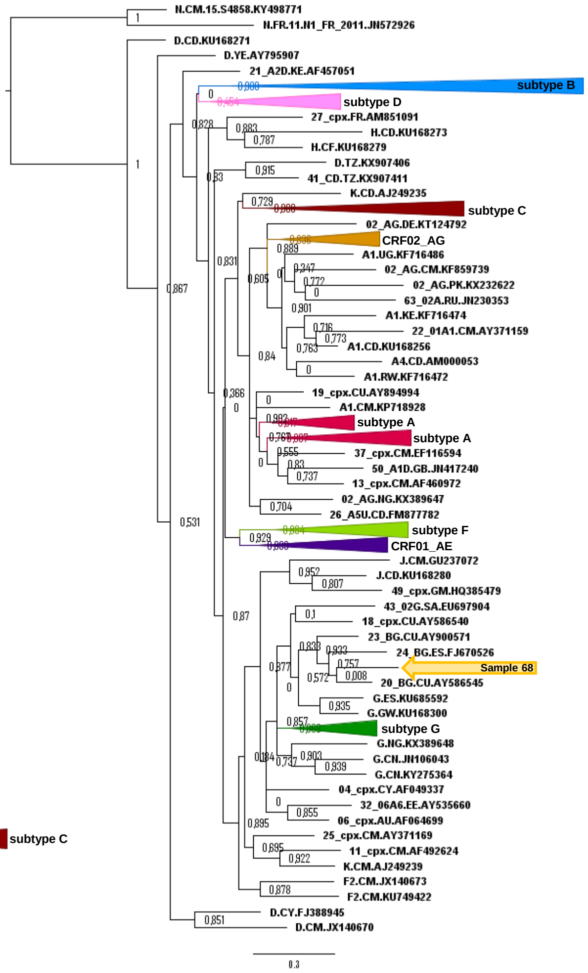

Sample 68: segment 12

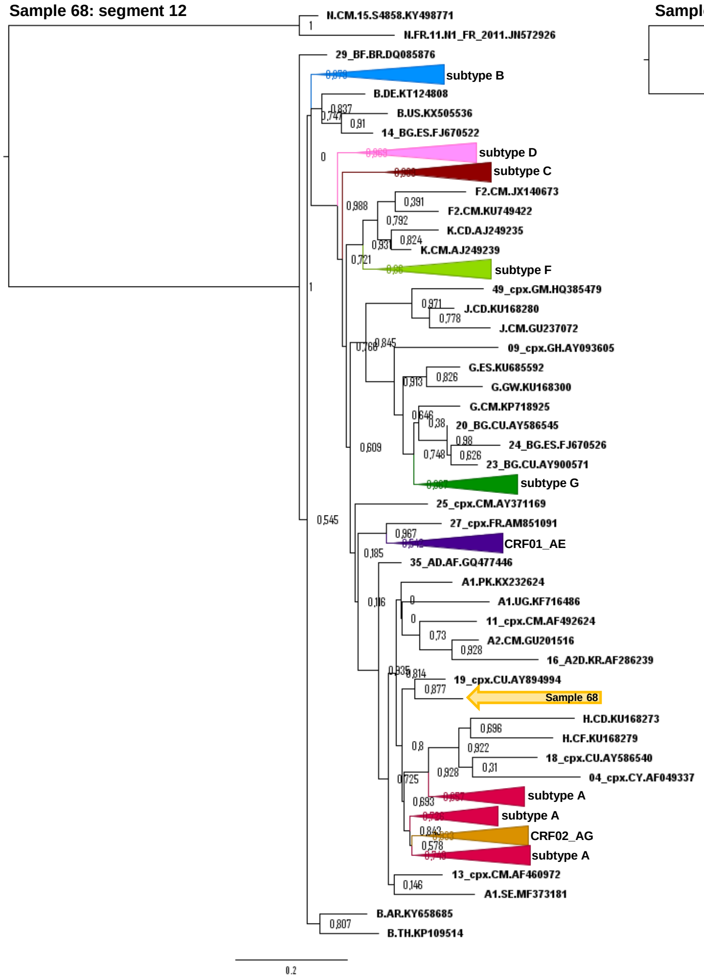

Sample 68: segment 13

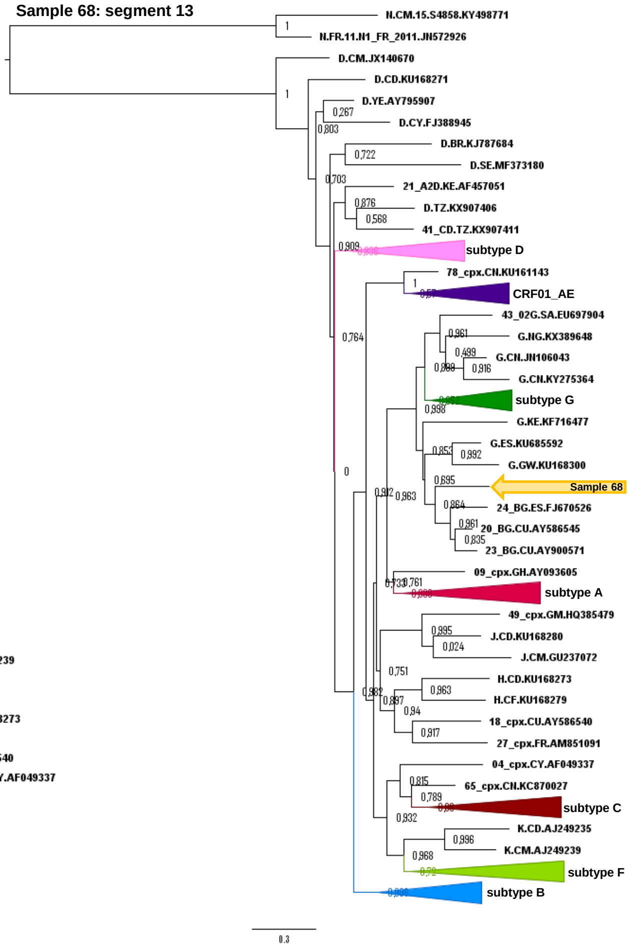

Sample 68: segment 14

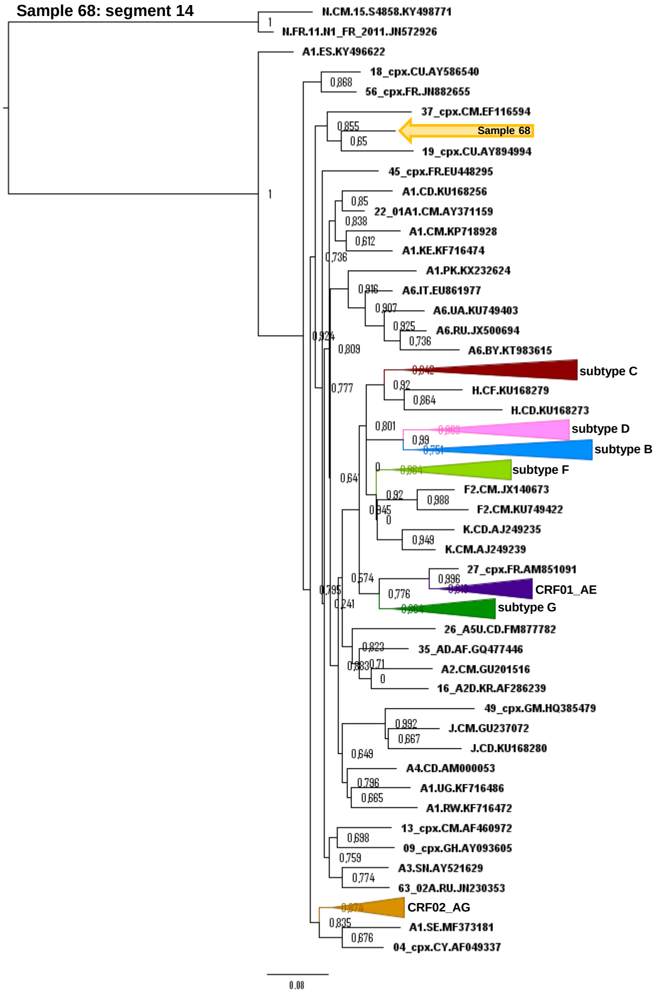

Sample 68: segment 15

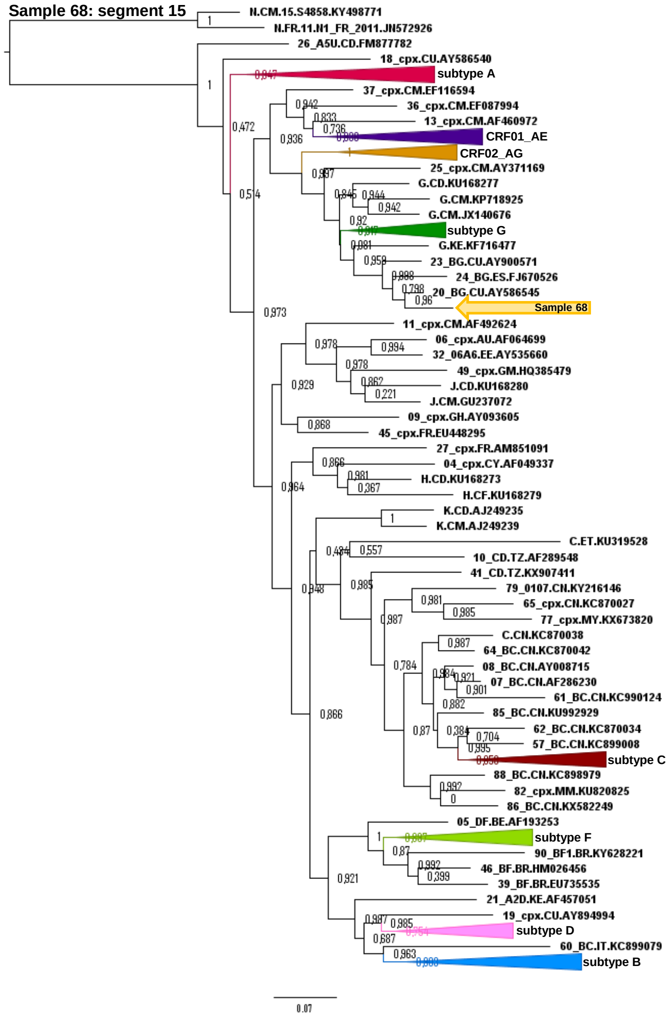

Supplement: Supplementary file 1 [file viruses-12-00063-s001.pdf]
